# Unraveling the habitat preferences, ecological drivers, potential hosts, and auxiliary metabolism of soil giant viruses across China

**DOI:** 10.1186/s40168-024-01851-8

**Published:** 2024-07-22

**Authors:** Jie-Liang Liang, Shi-wei Feng, Pu Jia, Jing-li Lu, Xinzhu Yi, Shao-ming Gao, Zhuo-hui Wu, Bin Liao, Wen-sheng Shu, Jin-tian Li

**Affiliations:** 1 Institute of Ecological Science, Guangzhou Key Laboratory of Subtropical Biodiversity and Biomonitoring, Guangdong Provincial Key Laboratory of Biotechnology for Plant Development, School of Life Sciences, South China Normal University, Guangzhou, 510631 People’s Republic of China; 2 School of Life Sciences, Sun Yat-Sen University, Guangzhou, 510275 People’s Republic of China

**Keywords:** Abundance–distribution relationship, Eukaryotic community, Geographic distribution, Soil nucleocytoplasmic large DNA viruses, Ecological drivers, Terrestrial ecosystem

## Abstract

**Background:**

Soil giant viruses are increasingly believed to have profound effects on ecological functioning by infecting diverse eukaryotes. However, their biogeography and ecology remain poorly understood.

**Results:**

In this study, we analyzed 333 soil metagenomes from 5 habitat types (farmland, forest, grassland, Gobi desert, and mine wasteland) across China and identified 533 distinct giant virus phylotypes affiliated with nine families, thereby greatly expanding the diversity of soil giant viruses. Among the nine families, *Pithoviridae* were the most diverse. The majority of phylotypes exhibited a heterogeneous distribution among habitat types, with a remarkably high proportion of unique phylotypes in mine wasteland. The abundances of phylotypes were negatively correlated with their environmental ranges. A total of 76 phylotypes recovered in this study were detectable in a published global topsoil metagenome dataset. Among climatic, geographical, edaphic, and biotic characteristics, soil eukaryotes were identified as the most important driver of beta-diversity of giant viral communities across habitat types. Moreover, co-occurrence network analysis revealed some pairings between giant viral phylotypes and eukaryotes (protozoa, fungi, and algae). Analysis of 44 medium- to high-quality giant virus genomes recovered from our metagenomes uncovered not only their highly shared functions but also their novel auxiliary metabolic genes related to carbon, sulfur, and phosphorus cycling.

**Conclusions:**

These findings extend our knowledge of diversity, habitat preferences, ecological drivers, potential hosts, and auxiliary metabolism of soil giant viruses.

Video Abstract

**Supplementary Information:**

The online version contains supplementary material available at 10.1186/s40168-024-01851-8.

## Background

Nucleocytoplasmic large DNA viruses (NCLDVs, also known as giant viruses) comprise an expansive group of eukaryotic viruses with remarkably large genomes ranging from about 100 kilobases to more than several megabases [1]. The largest known giant viruses even surpass numerous bacteria and archaea in both particle and genome size [1]. NCLDVs have been identified in diverse ecosystem components, including water, soil, vertebrate, and even human [2–5]. A growing number of NCLDVs have been reported to infect various eukaryotic lineages, especially phytoplankton groups and non-photosynthetic organisms in the ocean [6, 7]. Moreover, NCLDVs are increasingly believed to exert crucial ecological roles by accelerating the turnover of their hosts, changing eukaryotic community structure, and/or influencing biogeochemical cycles [4]. Therefore, NCLDVs have become of great interest owing to their large genomes, wide geographic distribution, and potential interactions with a wide range of eukaryotes [8].

In the last two decades, the discovery of new NCLDVs has been driven largely by co-cultivation with amoebae [3] or isolation together with their native hosts, such as the marine flagellate *Cafeteria roenbergensis*-infecting virus and the kinetoplastid-infecting Bodo saltans virus [9, 10]. Only recently, cultivation-independent methods such as metagenomics and single-cell genomics, have accelerated the discovery of new NCLDV members [11–13]. Remarkably, 501 and 2074 novel metagenome-assembled genomes (MAGs) of NCLDVs have been obtained by two recent studies from various ecosystems across the globe, which led to more than 10-fold expansion in phylogenetic diversity as well as functional diversity of NCLDVs [11, 13]. In a more recent study, Aylward et al. collected all giant virus genomes/MAGs available in NCBI RefSeq and in several publications and then selected 1380 dereplicated quality-checked genomes to perform a comprehensive phylogenetic analysis of giant viruses [14]. Their work uncovered that these giant viruses can be partitioned into six orders, 32 families, and 344 genera, substantially expanding our knowledge of the phylogenetic diversity of giant viruses [14]. Note, however, that in the abovementioned studies, most of the giant viruses (> 99%) were from marine and freshwater environments.

Besides the application of genome-based approaches, marker gene-based surveys have also been conducted to explore NCLDVs in the environment. Up till now, the most commonly used marker genes are the family B DNA polymerase (*polB*) and major capsid protein (MCP) genes. For instance, the populations of two NCLDV lineages (i.e., *Phycodnavirus* and *Mimivirus*) and their distinct geographical distributions in freshwater environments were characterized by sequencing of *polB* and/or MCP genes [15, 16]. Additional two previous studies, based on analysis of those *polB* sequences recovered from 18 and 283 metagenomes generated by Tara Oceans (an international multidisciplinary scientific program aiming to characterize ocean plankton diversity), illustrated that marine NCLDVs were highly diverse, showed a heterogeneous distribution across oceans, and likely had tight interactions with their potential hosts—microeukaryotes [17, 18]. Recently, a survey of MCP genes recovered from public metagenomes available in the IMG/M database revealed that NCLDVs were predominantly found in marine (approximately 55%) and freshwater (40%) environments, and to a much lesser extent in terrestrial (less than 1%) environments [13].

Whilst considerable progress has been made in recent years in understanding diversity, geographic distribution, and potential hosts of NCLDVs in aquatic environments, little is known about the biogeography and ecology of soil giant viruses. In a first attempt to explore NCLDVs in soil using cultivation-independent approaches, 13 novel and 3 previously known giant virus genomes were recovered from the metagenomes of soils from a forest ecosystem [12]. However, they represented only a tiny fraction of soil giant virus diversity in the ecosystem, given that nearly 245 MCP genes have been recovered from the unbinned metagenomic fragments [12]. Another study explored permafrost NCLDV diversity through metagenomics, revealing large genomic sequences of unknown families [19]. These findings suggest that soil giant viruses warrant further investigation [10]. Surprisingly, to the best of our knowledge, there has been no other work explicitly attempting to investigate NCLDVs in soil with metagenomics.

**Fig. 1:**
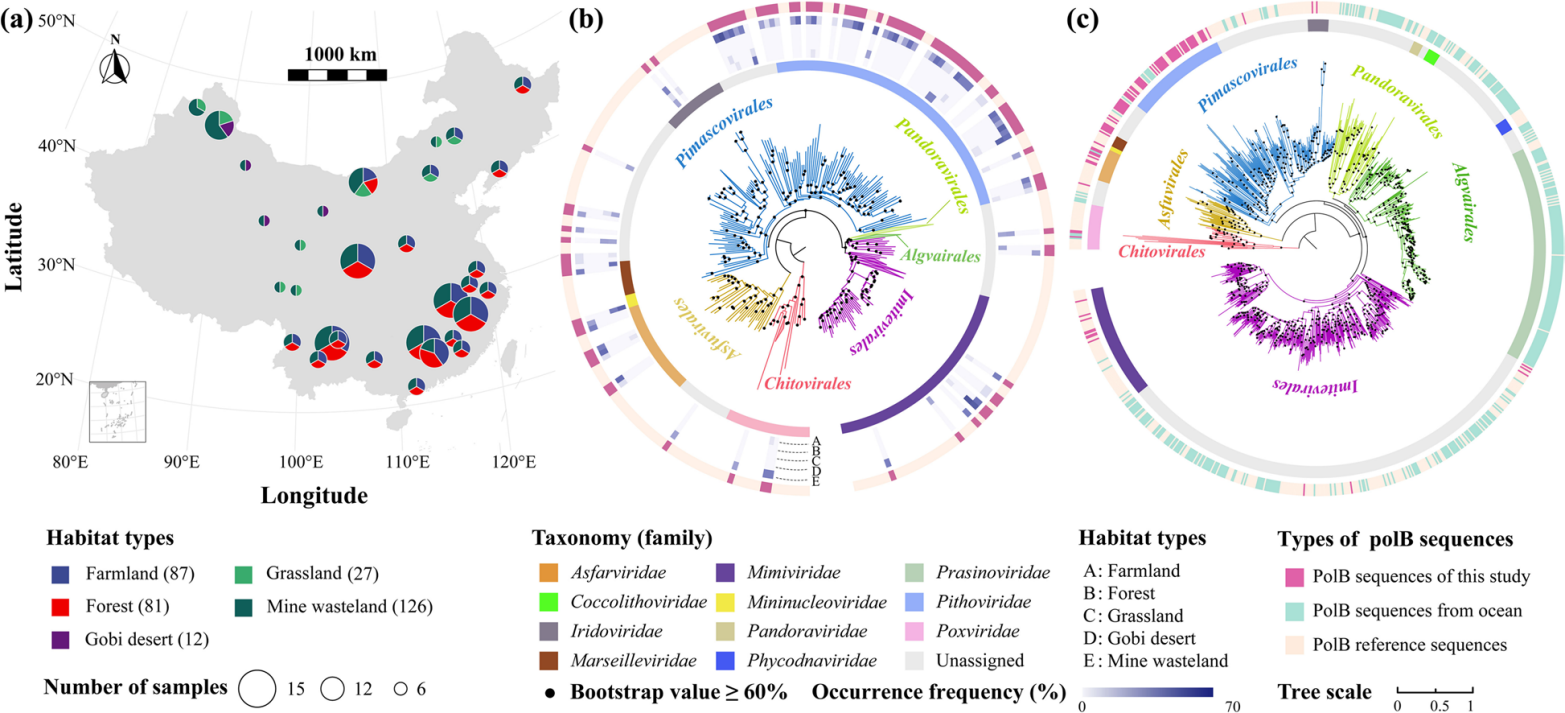
Sampling sites, phylogenetic affiliations, and occurrence frequencies of soil nucleocytoplasmic large DNA viruses (NCLDVs). **a** Geographic illustration of soil sampling sites on the map of China. The numbers in parentheses represent sample sizes for individual habitat types. Detailed information on each sampling site is provided in Supplementary Table S1. **b** Phylogenetic affiliations of soil NCLDVs and their occurrence frequencies (%) in each habitat type. Phylogenetic tree was constructed with 88 long (≥ 700 amino acid) PolB sequences from 333 soil metagenomes in this study and 502 PolB reference sequences. Tree branches are colored according to the order-level taxonomic assignment. The inner layer denotes the six well-recognized (*Asfarviridae*, *Iridoviridae*, *Marseilleviridae*, *Mimiviridae*, *Phycodnaviridae*, and *Poxviridae*) and five newly proposed (*Coccolithoviridae*, *Mininucleoviridae*, *Pandoraviridae*, *Pithoviridae* and *Prasinoviridae*) NCLDV families [14]. The middle five layers denote occurrence frequencies of individual NCLDV phylotypes in the five different habitat types labeled with A–E, respectively. The most outside layer denotes PolB reference sequences (orange) and PolB sequences recovered from this study (pink). **c** Phylogenetic tree of soil and marine NCLDV PolBs. Phylogenetic tree was constructed from 88 long PolB sequences from this study, 406 PolB sequences from a marine dataset [17], and 502 PolB reference sequences [14]. Tree branches are colored according to the order-level taxonomic assignment. The inner layer denotes the six well-recognized and five newly proposed NCLDV families as those in **b**. The outside layer denotes references (orange), soil (pink) and marine (blue) PolB sequences

In this study, we used *polB* as a marker gene to characterize NCLDVs in 333 soil metagenomes from 29 farmland, 27 forests, 9 grassland, 4 Gobi desert, and 42 mine wasteland ecosystems across 22 provinces of China (Fig. 1a and Supplementary Table S1). We identified a total of 533 distinct *polB* phylotypes, which were affiliated with nine giant virus families with cultivated representatives and considerably expanded the diversity of soil giant viruses. Moreover, we revealed the habitat preferences, ecological drivers, and potential hosts of the observed soil giant viruses. We further recovered 44 medium- to high-quality giant virus genomes from our metagenomes and uncovered their novel auxiliary metabolic genes. Overall, these findings shed new light on the biogeography and ecology of soil giant viruses.

## Methods

### A country-scale sample collection

A nationwide field survey was conducted between July and August 2018, wherein 42 study sites located across 22 provinces of China were involved (Fig. 1a). The study sites were selected to comprehensively represent the climatic, geographic, and edaphic features of China. At each study site, two to four types of terrestrial habitats were chosen for sampling when they were simultaneously distributed within an area of approximately 25 km^2^. Thus, the following five different types of terrestrial habitats were included in our survey: farmland, forest, Gobi desert, grassland, and mine wasteland. Specifically, a total of 333 soil samples were collected from 29 farmland, 27 forests, 4 Gobi desert, 9 grasslands, and 42 mine wasteland ecosystems (Fig. 1a and Supplementary Table S1). At each ecosystem, we collected three soil samples at a depth of 0 − 20 cm according to the method described previously [20]. The geographical coordinates like the longitude (LON), LAT, and ALT of each focal ecosystem were recorded using the Global Positioning System while sampling. Mean annual temperature (MAT) and MAP for each focal ecosystem were derived from the WorldClim Global Climate Database (http://worldclim.org). After the soil samples were transported back to the laboratory on ice, each soil sample was divided into two parts. One part was air-dried, ball-milled, sieved, and homogenized for physicochemical analyses; the other was stored at − 80 °C for DNA extraction.

### Physicochemical analysis

Soil pH was measured in a 1:2.5 (w/v) aqueous solution using a pH meter (Brand, Germany). Soil EC was measured by a DDS-307 conductometer (Shanghai, China). EX-Ca was extracted by CH_3_COONH_4_ solution and then measured by atomic absorption spectroscopy as described elsewhere [21]. Other soil physicochemical properties, including moisture, CEC, total carbon (TC), total N (TN), total P (TP), and total K (TK) were measured using standard methods [22]. Soil clay percentage (diameter < 0.002 mm) was determined by the method of soil particle-size fractionation [23].

### DNA extraction, PCR amplification and sequencing

Soil DNA was extracted using the FastDNA Spin kit (MP Biomedicals, Santa Ana, CA, USA) following the manufacturer’s protocol. DNA quality was assessed with the NanoDrop 2000 spectrophotometer (Thermo Scientific, USA). Each of the 333 high-quality soil DNA samples was divided into two parts. One part was used for whole metagenome sequencing to characterize NCLDVs, and the other part was used for 18S metabarcoding to explore the potential associations of soil eukaryotes with NCLDVs.

Each DNA sample for whole metagenome sequencing was further purified and used to construct a sequencing library (~ 300 bp average insert size). Subsequently, it was shotgun-sequenced on the Illumina HiSeq 2500 platform with PE150 mode (Illumina, USA). Detailed information on metagenomics data was provided in Supplementary Table S2. 18S rRNA genes were amplified using the eukaryotic primers 528F (5′-GCGGTAATTCCAGCTCCAA-3′) and 706R (5′-AATCCRAGAATTTCACCTCT-3′) spanning the V4 hypervariable region [24]. The amplicons were purified using resin columns (Qiagen, USA), checked for quality by NanoDrop 2000 spectrophotometer, and subsequently sequenced on the Illumina NovaSeq PE250 platform (Illumina, USA).

### Recruitment and taxonomic classification of *polB* genes

Raw reads filtering, contig assembly, and gene prediction were performed as described previously [25]. We recruited *polB* sequences from our 333 soil metagenomes according to Endo et al. [17]. Firstly, protein sequences were searched against a published hidden Markov model (HMM) of PolB sequences [17] using hmmsearch (version 3.2) [26] with *e* value of 10^−5^. In brief, the reference PolBs from the NCLDV orthologous gene cluster NCVOG0038 [27] were aligned using MAFFT-*linsi* [28], and the PolB HMM profile was constructed from the resulting alignment using hmmbuild [26]. The identified *polB* sequences were then dereplicated and clustered with CD-HIT (version 4.8.1) under the criteria of identity > 95% and overlap > 90% [17]. To remove those sequences not derived from NCLDVs, phylogenetic mapping was performed by maximum likelihood using a reference PolB phylogenetic tree.

Specifically, 502 representative NCLDV PolBs and 144 PolBs from eukaryotes, bacteria, archaea, and phages were selected as references [14, 17]. Of note, the 502 NCLDV PolB references represented all six currently known NCLDV orders (*Algavirales*, *Asfuvirales*, *Chitovirales*, *Imitervirales*, *Pandoravirales*, and *Pimascovirales*) and all 32 currently known NCLDV families, including six well-recognized (i.e., *Asfarviridae*, *Marseilleviridae*, *Iridoviridae*, *Mimiviridae*, *Phycodnaviridae,* and *Poxviridae*) and five newly proposed families (i.e., *Coccolithoviridae*, *Mininucleoviridae*, *Pandoraviridae*, *Pithoviridae*, and *Prasinoviridae*) with cultivated representatives as well as 20 newly proposed families with only one or none cultivated representative [14]. The 646 reference PolB sequences were aligned together using MAFFT with *linsi* option (version 7.490) [28], and the maximum likelihood tree was constructed using RAxML with the parameters -m PROTGAMMAWAG -p 23333 (version 8.2.12) [29].

To identify NCLDV PolBs, the query sequences were first aligned against the reference alignment using MAFFT with “addfragments” option, and then mapped onto the reference tree using pplacer (version 1.1.alpha19) [30]. A sequence was identified as a giant virus if it was placed within a given NCLDV clade. To test the robustness of the NCLDV PolB identification pipeline, we selected 612 PolB sequences of eukaryotes (111), bacteria (244), archaea (226), and phages (31), which included the representative sequences of the known 20 PolB groups presented in previous work (for sequence information, please see Supplementary Table S3) [31]. We subjected these 612 sequences to our pipeline and found that none of them passed the filtering (i.e., none of them were erroneously identified as NCLDV PolBs).

Our PolBs were then subjected to the above pipeline for identification. Taxonomic classification of our NCLDV PolBs was performed as per Aylward et al*.* [14]. All of our sequences could be assigned to the six known orders. The sequences placed within the six well-established and five newly proposed families with known cultivated representatives were assigned to the corresponding families, and those placed basal to these families were defined as unassigned families. Other sequences clustered within or basal to the 20 newly proposed families with one or no cultivated representative were also defined as unassigned families. It has been reported previously that frequent *polB* gene transfers and lack of adequate references in *Pandoravirales* might cause many long branches in the pandoravirales clade, making phylogenetic reconstruction of this group rather difficult [14]. After careful manual inspection, sequences affiliated with *Pandoravirales* were removed from further analysis for the long branches in the pandoravirales clade and relatively low bootstrap values (< 75%). Consequently, a total of 533 non-redundant NCLDV *polB* genes were retained for subsequent analyses. For a better comparison between soil and oceanic NCLDVs, the NCLDV *polB* genes of Endo et al. [17] were re-classified using the abovementioned method.

### Abundance profiling of *polB* genes

To get *polB* gene coverage information, high-quality reads from each metagenome were individually mapped to each *polB* gene using BBMap v38.44 with the parameters *k* = 14, minid = 0.97, and build = 1. The normalized gene coverage table was generated as described elsewhere [32]. Briefly, the *polB* coverage in a given metagenome was first divided by the reads number of that metagenome and then multiplied by the average of the reads number of the 333 metagenomes. Relative abundance profiles of all *polB* genes were then generated by transferring the normalized coverage table to the proportional table (sum to 100% within each metagenome).

### Mapping the identified *polB*s at a global scale

To project the distribution of the *polB* genes identified in this study onto a global scale, a total of 288 global topsoil metagenomes were downloaded from the European Bioinformatics Institute Sequence Read Archive database (PRJEB24121) [33]. Raw reads were processed as described previously [25] and then reads shorter than 175 bp were removed [33]. High-quality reads from each metagenome were separately mapped to each *polB* gene of this study using BBMap with the abovementioned parameters.

### Phylogenetic analysis of PolB proteins

Long PolB sequences (≥ 700 amino acid sequences) were selected to construct the phylogenetic trees, because short sequences may yield unreliable phylogeny [17]. The selected 88 protein sequences were aligned with 502 known NCLDV PolBs [14, 17] using MAFFT [28]. The maximum likelihood tree was constructed using IQ-TREE (version 2.1.2) with ultrafast bootstraps calculated (-m TEST, -bb 1000, -alrt 1000, –bnni) [34]. The PolB tree was visualized using the Interactive Tree of Life online interface [35].

### Diversity analysis of NCLDVs

The analyses of α diversity (richness and Shannon index) and β diversity were performed based on the normalized coverage table using the vegan package in R software (version 4.1.0). Specifically, the NCLDV richness data was obtained by counting the number of PolB sequences in each metagenome, and significant differences among habitat types in NCLDV richness were assessed using the Kruskal–Wallis test.

The β diversity was analyzed by NMDS ordinations based on the Bray–Curtis dissimilarity matrices. Permutational multivariate analysis of variance (PERMANOVA; 999 permutations) was used to test for statistically significant differences among habitat types in NCLDV community composition.

### Assessment of eukaryotic diversity

Raw paired-end 18S reads were denoised, dereplicated, quality filtered, and merged via the Quantitative Insight into Microbial Ecology (QIIME2) platform following the DADA2 pipeline [36]. Reads were trimmed into 208 bp for forward reads and 206 bp for reverse reads according to quality scores. Error-free and non-chimeric amplicon sequencing data were then processed into amplicon sequence variants (ASVs, a higher-resolution substitute of operational taxonomic units) using the QIIME2 pipeline, and singletons were discarded. Eukaryotic taxonomic assignment was carried out using classify-sklearn provided by the q2-feature-classifier plugin against SILVA database (v138) pretrained with naïve Bayes algorithm [37]. We used the QIIME2 core-metrics plugin to produce the rarefied sequence-variant Table (43,196 for 18S rRNA sequences). On the basis of the rarefied feature table, the Shannon index was calculated as described above and the observed number of eukaryotic ASVs in a given soil sample was used to represent the eukaryotic richness of that sample. The compositional variations of eukaryotic communities of soil samples were assessed by the Bray–Curtis dissimilarity matrix with the vegan package.

### Correlation analysis

All statistical analyses in this study were performed using specific packages in R software (version 4.1.0). First- and second-order polynomial fits were performed to investigate the main determinants of NCLDV diversity in different habitat types. We selected prominent biotic and abiotic variables that we expected to have significant effects on NCLDV diversity on the basis of previous studies [33, 38]. However, there was strong collinearity among particular environmental factors, and we used variable clustering to assess the redundancy of the environmental variables by “varclus” procedure using Hmisc package [39]. The variables with higher correlation (Spearman’s *ρ*^2^ > 0.7) were removed from the regression analysis. The resulting 14 environmental variables (including LON, LAT, ALT, MAP, pH, EC, EX-Ca, CaCO_3_, CEC, clay, TP, TK, TN, and TC; Supplementary Table S1) and eukaryotic richness index were used to assess their correlations with α diversity of NCLDVs using a regression model. Prior to model selection, variables were evaluated for linearity and multicollinearity. The degree of polynomial functions (linear, quadratic) was chosen on the basis of the lowest Akaike Information Criterion values with basicTrendline package.

### Calculation of environmental ranges of NCLDV phylotypes

The environmental range of a given phylotype is defined as the average breadth of certain environmental conditions where that phylotype is present [40]. In this study, the environmental range was calculated based on the abovementioned 14 environmental variables (Supplementary Table S1) according to Barberan et al. [40]. Briefly, individual variables for each sample were standardized from zero to one. Following this, the mean of the 14 standardized environmental variables (SEV) for each sample was calculated. The environmental range of a given phylotype was calculated by subtracting the minimum SEV from the maximum SEV for that phylotype, which was then standardized from zero to one.

### Random forest analysis

Random forest analysis was used to identify the relative contributions of the above-mentioned 14 environmental factors and eukaryotic diversity in predicting NCLDV α diversity of different habitat types using randomForest and rfPermute package [41]. To avoid fitting bias and to ensure model simplicity, we excluded the variables that had no significant effects on NCLDV α diversity. That is, the backward selection would be stopped if there was no model improvement seen in the variation being explained (*R*^2^) or if the whole model and variables were all significant (*P* < 0.05). After the best fitting Random forest models were obtained, the significance of the models was assessed and *R*^2^ with default parameters of response variable was cross-validated using the A3 package.

### Variation partition analysis (VPA)

VPA was conducted to quantify the relative contributions of climatic (MAP), geographical (LON, LAT, and ALT), physicochemical factors (pH, EC, EX-Ca, CaCO_3_, CEC, clay, TP, TK, TN, and TC), and eukaryotic community composition to the variations in β diversity of NCLDVs, using function “varpart” in vegan package. Principle components analysis was performed before VPA to reduce the dimensionality of physicochemical factors and eukaryotic community composition.

### Partial Mantel test

A partial Mantel test was performed to evaluate the correlations between two multivariate (abundance) matrices controlling either the potential effects of eukaryotic community compositions or geographic distances (spatial autocorrelation) using the vegan package. Abundance matrix for the NCLDV community was constructed from the normalized coverage table and that for the eukaryotic community was made from the re-sampled ASV abundance table. Distance matrices for NCLDV and eukaryotic communities were computed by the Bray–Curtis dissimilarity, while Euclidean distance was used for calculating the abiotic factors in each habitat type. The geographical distances among sampling sites were measured using the function “distm” in the geosphere package. Partial Mantel correlations based on Pearson correlation were computed between all pairs of distance matrices of NCLDVs, eukaryotes, and other potential influencing factors with 999 permutations for each comparison.

### Microbial co-occurrence network analysis

A co-occurrence network analysis was used to determine the potential relationships between NCLDVs and eukaryotes across the five habitat types. To remove poorly represented species, we included NCLDV phylotypes and eukaryotic ASVs that were present in ≥ 10% of all soil samples for each habitat type. The abundances of individual pairwise combinations of NCLDVs and eukaryotes across samples in every habitat type were calculated using Spearman’s rank correlation with *P* < 0.05 (adjusted by Benjamini and Hochberg linear step-up procedure to control false discovery rate) to infer a co-occurrence network [42]. Only those correlations that fell within the stringent cutoff (correlation coefficients ρ ≥ 0.60) were considered to be significant and included in the network [43]. Similarly, the 31 NCLDV phylotypes detected in all five habitat types were used to construct the co-occurrence network with eukaryotes. The co-occurrence networks were visualized by ggraph (version 2.0.5) and igraph (version 1.2.6) packages.

### Binning and analysis of NCLDV genomes

To generate giant virus metagenome-assembled genomes (GVMAGs), binning was performed according to a previously published protocol [13]. Briefly, contigs with length longer than 5 kb were used to screen putative NCLDV contigs by either of the following criteria: (1) classified as NCLDV-specific contigs by a published automatic classifier [13]; (2) containing at least 2 out of 20 ancestral nucleocytoplasmic virus orthologous groups (NCVOGs) [13]; (3) containing NCLDV *polB* gene (NCVOG0038). Putative NCLDV contigs were pooled and binned using MetaBAT2 with default parameters [44]. GVMAGs were then de-contaminated, de-duplicated, and quality-checked following Schulz’s protocol [13]. The taxonomy of GVMAGs was inferred by constructing phylogenetic trees together with known NCLDVs published by Aylward et al. [14]. The ORFs in the GVMAGs were predicted with MetaProdigal [45]. For functional annotation, all the ORFs were queried against the KEGG database using BLASTp, the VOG database using hmmsearch, and the Pfam database using InterProScan with an *E* value cut-off of 10^−5^. To identify carbohydrate-active enzyme genes, all predicted ORFs were searched against the dbCAN2 meta server with default parameters [46].

## Results

### Phylogenetic affiliations and occurrence frequencies of soil NCLDV phylotypes

We recovered a total of 533 distinct NCLDV *polB* genes (Supplementary Table S4) from our 333 soil metagenomes. The percentage (32.3%) of NCLDV *polB* sequences ≥ 1 kbp, the median length (750 bp), and the mean length (1232 bp) of our NCLDV *polB* sequences were comparable to those (32%, 738 bp, and 1117 bp, correspondingly) of NCLDV *polB* sequences from the global seawater samples (Supplementary Fig. S1) [17].

The NCLDV phylotypes in this study were affiliated with five orders, including *Algavirales*, *Asfuvirales*, *Chitovirales*, *Imitervirales*, and *Pimascovirales* (Fig. 1b and Supplementary Table S4). Among the 301 phylotypes that could be assigned to the nine known NCLDV families with cultivated representatives, the number of phylotypes was highest in *Pithoviridae* (109), followed by *Mimiviridae* (81), *Asfarviridae* (45), *Iridoviridae* (23), *Poxviridae* (22), *Marseilleviridae* (11), *Mininucleoviridae* (6), *Prasinoviridae* (2), and *Phycodnaviridae* (2). In order to compare the identified soil NCLDVs with marine NCLDVs, we also inferred the taxonomic affiliations of the 6523 NCLDV phylotypes obtained from the global Tara Oceans metagenomes datasets (Supplementary Table S5) [17]. At the family level, the phylotypes of *Pithoviridae*, *Asfarviridae*, and *Marseilleviridae* were mainly from soil samples, whilst those of *Prasinoviridae* were mostly from the ocean (Supplementary Tables S4 and S5 and Fig. 1c).

**Fig. 2:**
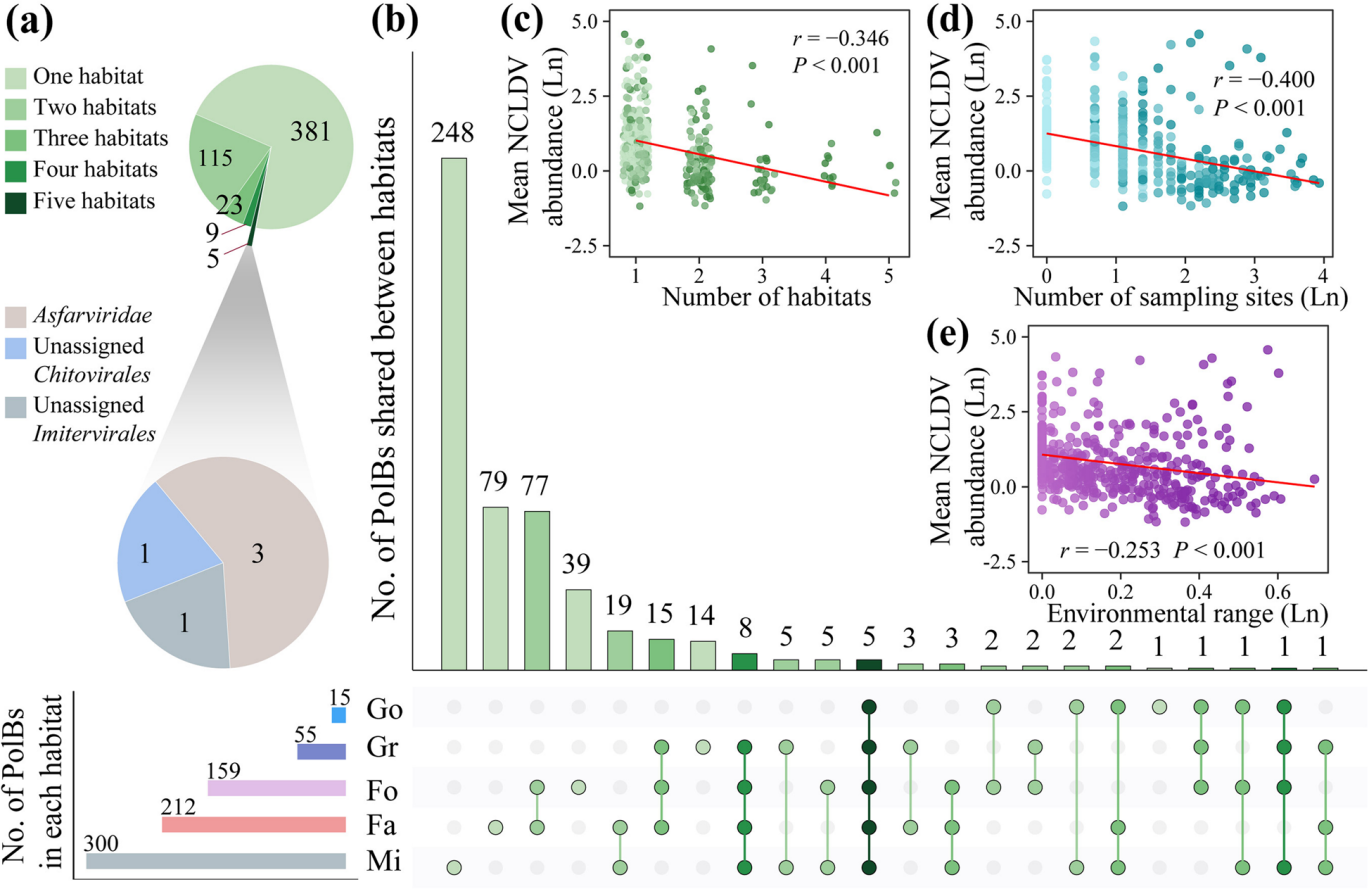
Ubiquity, uniqueness, and abundance–distribution relationships of soil NCLDVs. **a**, **b** Shared and unique NCLDV phylotypes of the five different habitat types. Fa, farmland; Fo, forest; Mi, mine wasteland; Gr, grassland; Go, Gobi desert. **c**–**e** Correlations between the abundances of individual NCLDV phylotypes and the numbers of habitat types where they could be recovered (**c**), the numbers of sampling sites where they could be recovered (**d**), and their environmental range (**e**). Environmental range was calculated as the mean of the ranges of individual environmental factors standardized from zero to one according to the method of Barberan et al. [45]. Abundance, the numbers of sampling sites, and environmental range are normalized by logarithm

Mine wasteland harbored as many as 300 NCLDV phylotypes, followed by farmland (212), forest (159), grassland (55), and Gobi desert (15; Fig. 2b). Rarefaction analysis showed that at the end of sampling the number of phylotypes in farmland, forest, and mine wasteland increased very slowly by 0.34–0.43% per sample (Supplementary Fig. S2). The heterogeneous distribution of soil NCLDVs across habitat types was further reflected by the varying occurrence frequencies of individual phylotypes (Fig. 1b and Supplementary Table S4). Some members of *Asfarviridae*, *Mimiviridae*, and *Iridoviridae* were found in at least four habitat types (Fig. 2 and Supplementary Table S4), whereas others occurred only in certain habitat types. More specifically, most phylotypes affiliated with *Pithoviridae* (94/109) and *Mimiviridae* (56/79) were mainly detected in mine wasteland, with occurrence frequencies of 1.3–12% and 1.3–15% respectively (Fig. 1b and Supplementary Table S4).

### Ubiquitous and unique soil NCLDV phylotypes across habitat types

We also analyzed the overlap and uniqueness of the NCLDV phylotypes identified in individual habitat types to further evaluate their habitat preferences (Fig. 2a, b). Only five phylotypes (0.94% of the total) were shared by five habitat types, however, over half of the total phylotypes of this study (381; 71%) were detected in only one habitat type. Remarkably, as many as 248 unique phylotypes were identified in mine wasteland, with a third of which (82; 33%) affiliated with *Pithoviridae*.

In order to get some insights into the observed habitat occupancy patterns of soil NCLDVs, we explored the relationships between the number of sites where individual phylotypes are present, the total/average abundance of individual phylotypes, and their environmental range. The number of sites and the total abundance were positively correlated with the environmental range (*P* < 0.001, Supplementary Fig. S3). In contrast, the average abundance was found to be negatively correlated with the number of habitat types, the number of sampling sites, and the environmental range (*P* < 0.001, Fig. 2c–e). Similar patterns were also observed for at least four families (*Asfarviridae*, *Marseilleviridae*, *Mimiviridae*, and *Mininucleoviridae*; Supplementary Fig. S4) when phylotypes from individual families were taken into separately.

**Fig. 3:**
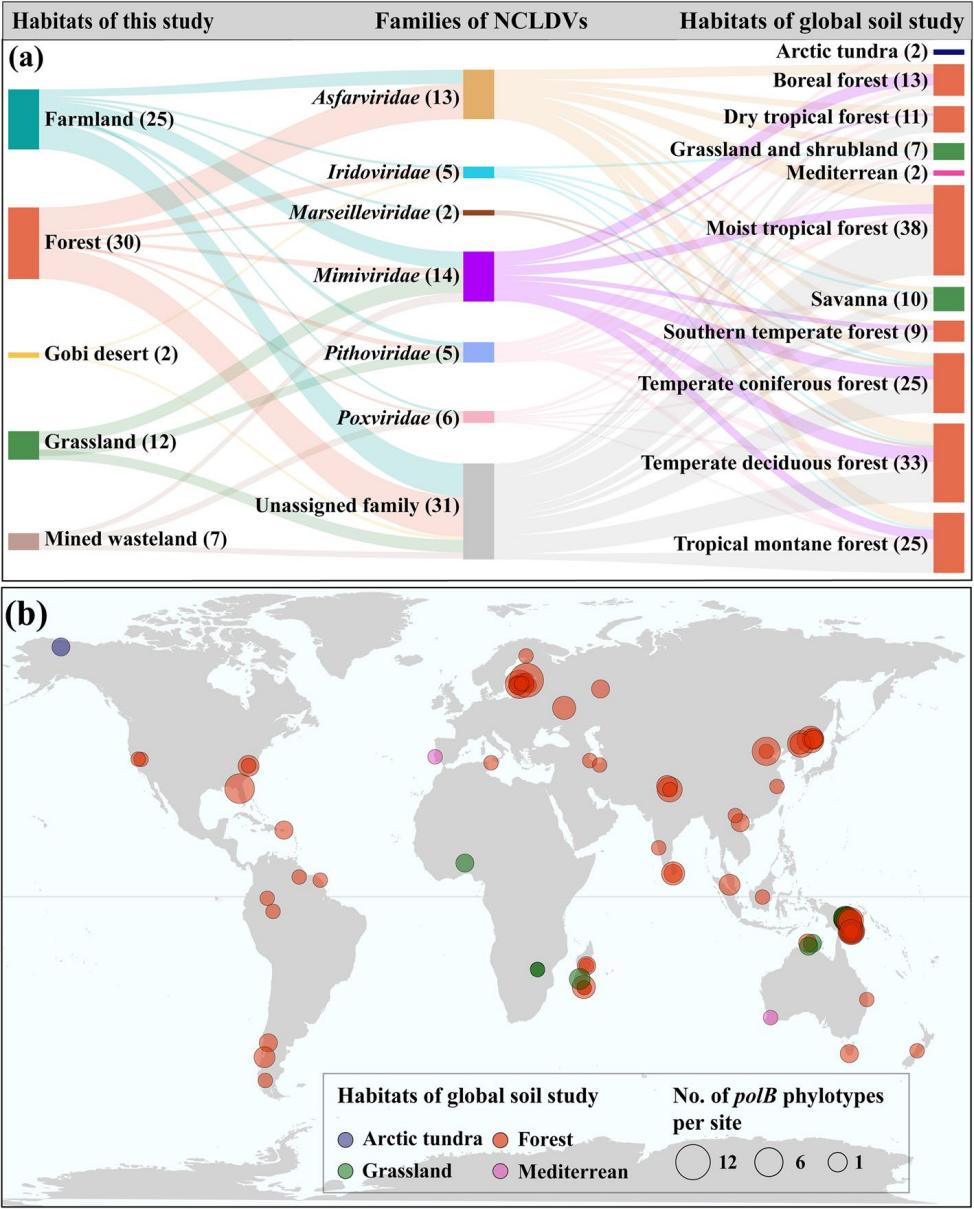
Global distribution patterns of the soil NCLDVs identified in this study. **a** Sankey flow diagram showing the habitat sources, quantities, and taxonomic affiliations of those NCLDVs that were not only identified in this study but also detectable in a published global topsoil metagenome dataset (‘global soil study’) [33]. Habitat types of this study and the global soil study are shown in different colors on the left and right, respectively. Taxonomic affiliations (families) of NCLDVs are shown in the middle. The heights of the individual bars are proportionate to the numbers of NCLDV phylotypes identified in different habitat types or belonging to various families, which are also presented in parentheses. The widths of the lines between habitat types and families represent the magnitudes of the shared NCLDV phylotypes. **b** Map showing the sampling sites of the global soil study and the numbers of NCLDV phylotypes detected in individual sampling sites. Circles represent the sampling sites and are colored based on habitat types. Circle sizes reflect the numbers of phylotypes detected in the corresponding sampling sites. Circles at the same coordinates are stacked according to their size, with the largest one at the bottom

Approximately 14% of the phylotypes (76/533) identified in this study were detectable in a previously published global topsoil metagenome dataset (> 61% of all the metagenomes; Fig. 3) [33]. These phylotypes were mainly recovered from our soil metagenomes of forest (30) and farmland (25; Fig. 3a). Among them, those of *Mimiviridae* were most dominant (14), accounting for 18% of the total detectable phylotypes. Among the 11 habitat types of the global topsoil study, moist tropical forests harbored the highest number of phylotypes (38). Remarkably, two forest soil samples were found to foster phylotypes affiliated with up to four families. All six continents investigated by the global soil study contained NCLDVs identified in this study, although the number of phylotypes varied greatly from 12 to 1 per site (Fig. 3b). Taken together, these results indicate the ubiquitousness of soil NCLDVs across the globe.

### Community compositions and α diversities of soil NCLDVs

**Fig. 4:**
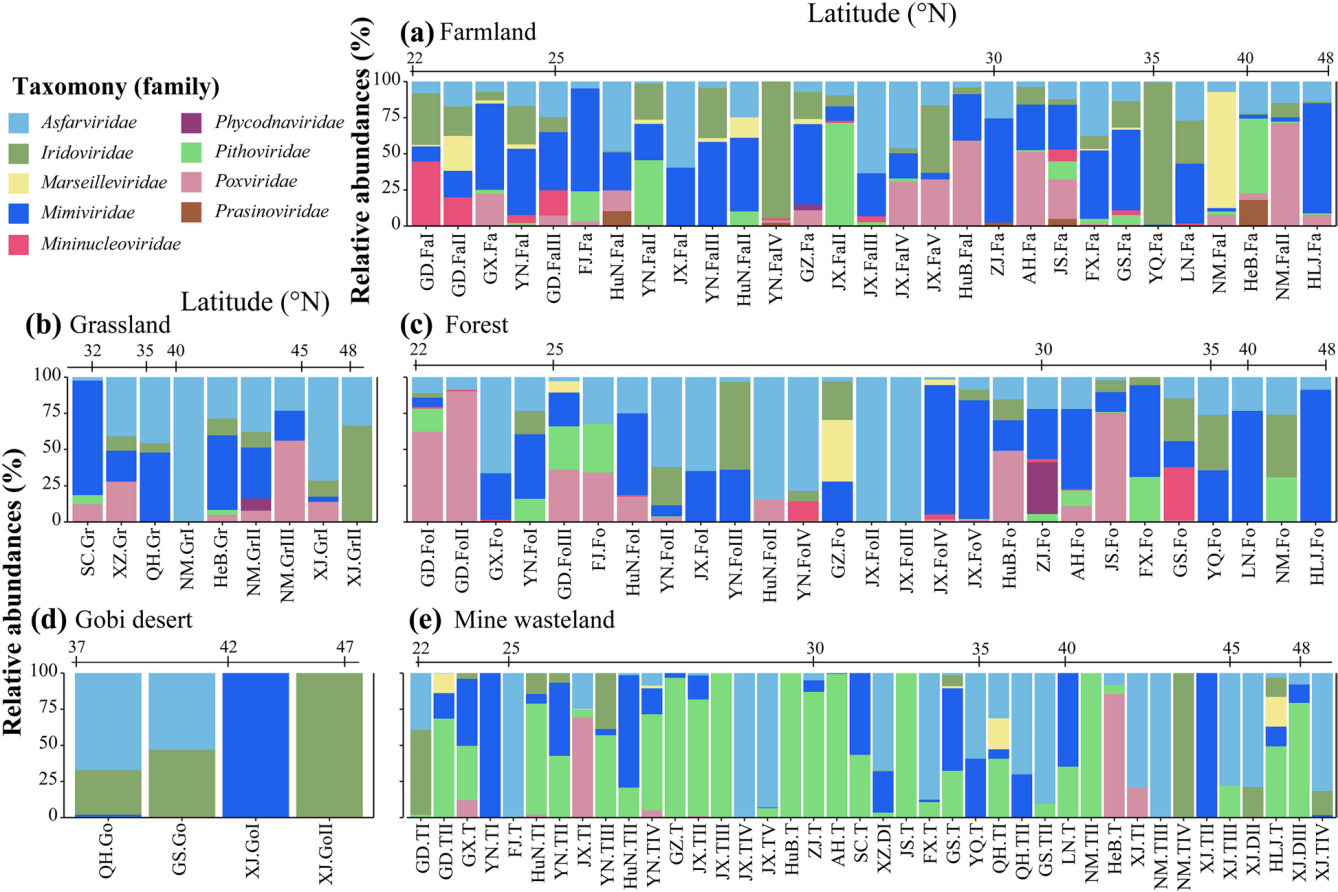
Community compositions and the numbers of soil NCLDVs in individual sampling sites. Relative abundances of various NCLDV families are shown in the bar charts. Sampling sites are first grouped as per their habitat types [farmland (**a**), forest (**b**), grassland (**c**), Gobi desert (**d**), and mine wasteland (**e**)] and then those within the same habitat type are arranged according to their latitudes (from south to north)

The community compositions of soil NCLDVs in individual ecosystems (sampling sites) of each habitat type varied greatly (Fig. 4). No single family was detected in all of the individual ecosystems of a given habitat type (except *Asfarviridae* in grassland), and the most dominant NCLDV families in individual ecosystems of a certain habitat type altered considerably. On average, *Asfarviridae* and *Mimiviridae* contributed to the total NCLDV abundance in the ecosystems of farmland, forest, and grassland in a range of 20–43% and 28–33%, respectively (Fig. 4a–c). In contrast, the most dominant family in mine wasteland was *Pithoviridae*, contributing to 37% of the total NCLDV abundance in all samples (Fig. 4e).

**Fig. 5:**
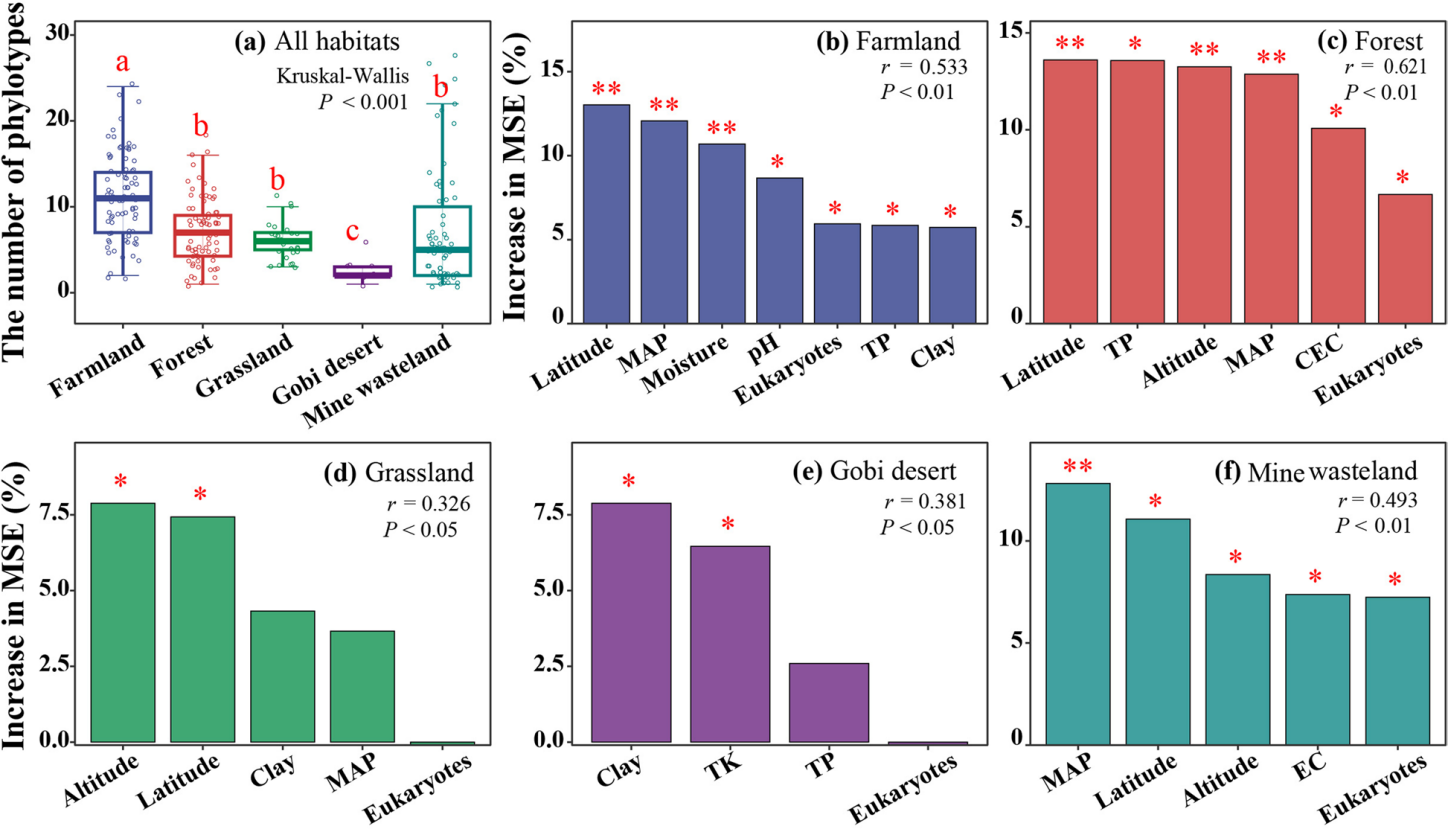
Alpha-diversities of soil NCLDVs in different habitat types and their major predictors. **a** NCLDV phylotype richness. Horizontal lines represent the medians, whereas the boxes represent the interquartile ranges of the first and third quartiles. The vertical lines represent the maximal and minimal values. Different letters on the top of the bars indicate significant differences between individual medians assessed with Kruskal–Wallis tests. **b**–**f** Major predictors of NCLDV phylotype richness in farmland (**b**), forest (**c**), grassland (**d**), Gobi desert and mine wasteland (**f**), respectively. The relative importance of selected predictors, quantified by an increase in the mean square error (MSE), was illustrated by random forest analysis. Significance levels of individual predictors are represented by * (*P* < 0.05) or ** (*P* < 0.01). LAT, latitude; ALT, altitude; MAP, mean annual precipitation; EC, electrical conductivity; EX-Ca, exchangeable calcium; CEC, cation exchange capacity; TC, total carbon; TN, total N; TP, total P; TK, total K; Eukaryotes, the number of eukaryotic amplicon sequence variants (ASVs)

The number of phylotypes was used to investigate the variations of NCLDV α diversities among different habitat types. The average number of phylotypes in farmland was significantly higher than those of the other four habitat types (*P* < 0.001, Fig. 5a). Random forest analysis identified altitude (ALT), latitude (LAT), and mean annual precipitation (MAP) as important predictors of α diversities in at least three habitat types (*P* < 0.05, Fig. 5b–f). Eukaryotic richness was also an important factor shared in farmland, forest, and mine wasteland. In order to explore how individual environmental variables affect NCLDV α diversity in different habitat types, correlation analyses were conducted for the number of NCLDV phylotypes and those variables with low autocorrelation (Supplementary Figs. S5 and S6). Remarkably, in farmland, forest, and mine wasteland, NCLDV α diversities peaked in the sampling sites with a MAP of approximately 1000 mm and declined towards those with lower or higher MAP (*P* < 0.01, Supplementary Fig. S6k, l, and o).

### β diversities of soil NCLDVs in different habitat types and their ecological drivers

**Fig. 6:**
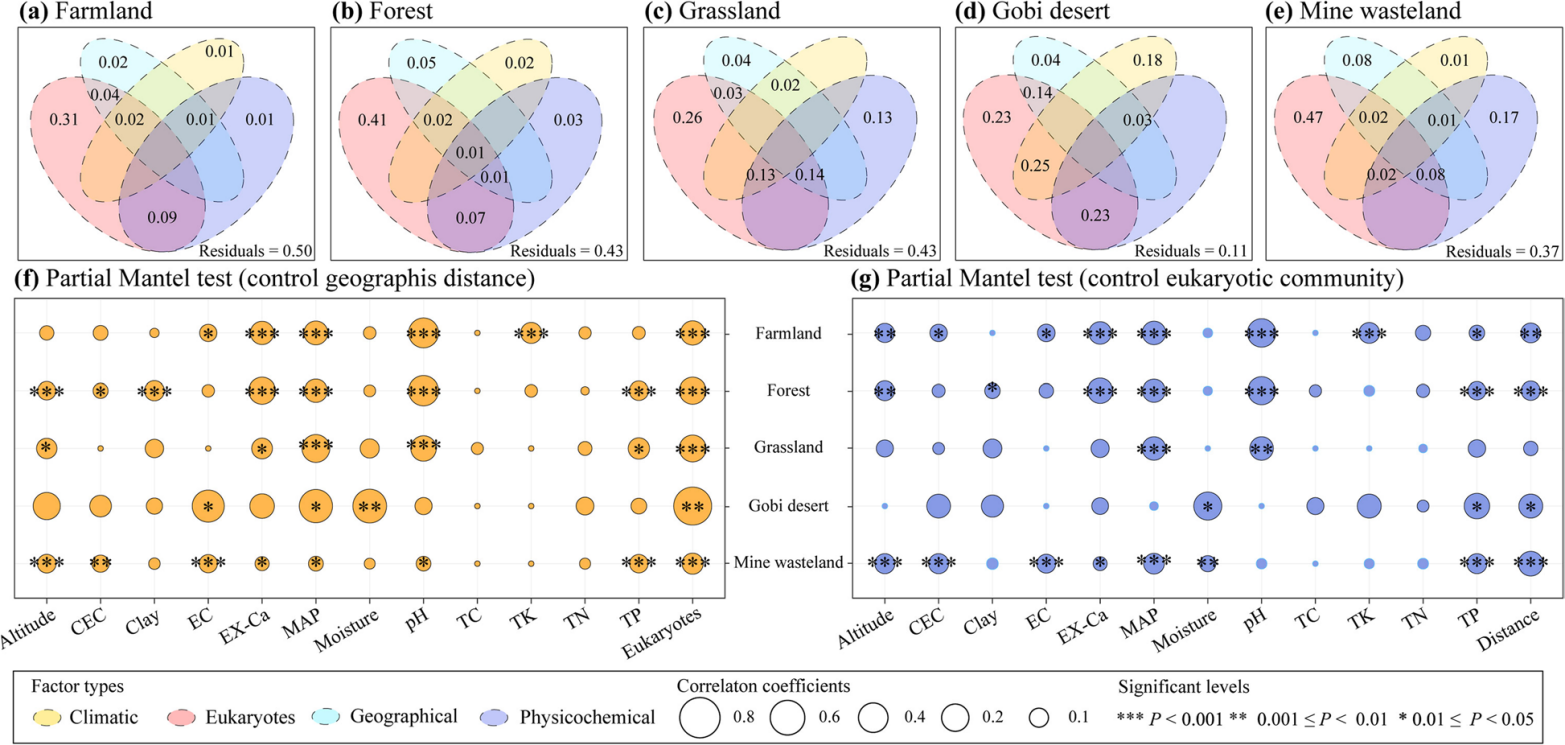
Major driving factors of beta-diversities of soil NCLDVs in different habitat types.** a**–**e** Variation partitioning analysis (VPA) differentiating effects of climatic, geographical, and physicochemical factors and eukaryotic community composition on NCLDV community composition in farmland (**a**), forest (**b**), grassland (**c**), Gobi desert (**d**) and mine wasteland (**e**). **f**, **g** Partial Mantel correlations (Spearman correlation coefficients) between NCLDV community composition and different ecological factors with controls for geographic distance (**f**) and eukaryotic community composition (**g**). Abbreviations are as those in Fig. 5

Non-metric multidimensional scaling (NMDS) analysis showed that the soil NCLDV communities of different habitat types differed considerably from each other (*P* = 0.001, Supplementary Fig. S7). VPA was performed to assess the relative importance of the climatic, geographical, and edaphic characteristics as well as eukaryotic community composition in explaining the variance of NCLDV communities in different samples within individual habitat types. As a whole, the four types of factors explained 50%, 57%, 57%, 89%, and 63% of the total variance in farmland, forest, grassland, Gobi desert, and mine wasteland, respectively (Fig. 6a–e). Among them, eukaryotic community composition was the most influential one, contributing to 31%, 41%, 26%, 23%, and 47% of the total variance in the five habitat types, correspondingly.

Partial Mantel test (after taking into account the effect of geographic distance) revealed significant relationships between NCLDV β diversity and eukaryotic community composition in all five habitat types (*P* < 0.01, Fig. 6f). In addition, MAP was also found to be significantly correlated with NCLDV β diversity in all five habitat types. Additionally, two other environmental variables (i.e., exchangeable calcium (Ex-Ca) content and pH) were shared by four habitat types. Despite this, the effect of geographic distance among samples cannot be negligible, given that (1) significant distance-decay relationships were observed for the NCLDV communities in all five habitat types (*P* < 0.01, Supplementary Fig. S8); and (2) partial Mantel test (after accounting for the effect of eukaryotic community composition) identified significant relationships between NCLDV β diversity and geographic distance in four habitat types except grassland (*P* < 0.05, Fig. 6g).

### Associations between soil NCLDVs and eukaryotes in different habitat types

**Fig. 7:**
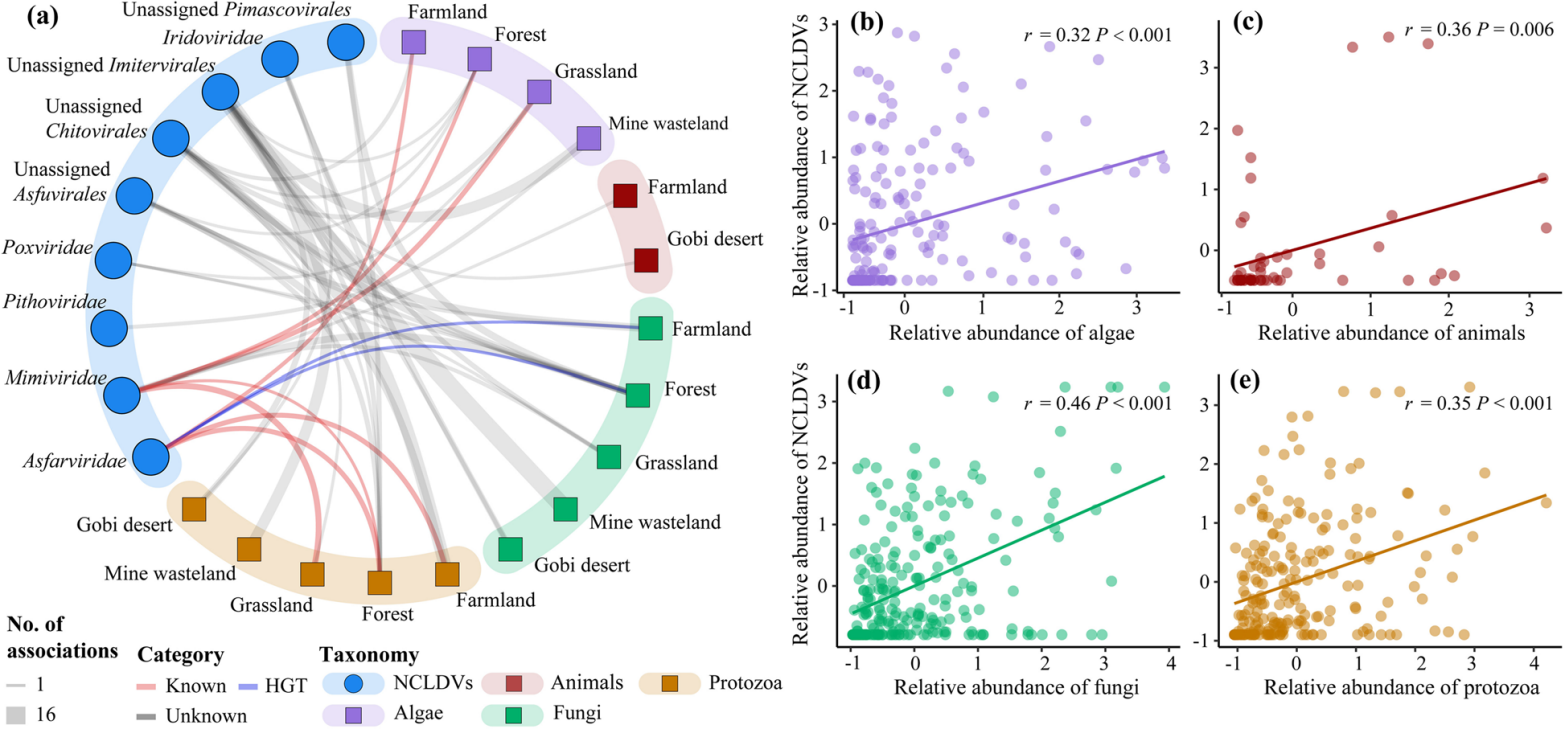
Associations of NCLDVs–eukaryotes in different habitat types. **a** Summary of the co-occurrence networks of NCLDVs–eukaryotes in five habitat types illustrated in Supplementary Fig. S9. Circles represent NCLDV phylotypes and squares represent eukaryotic ASVs present in certain habitat types. The numbers of associations between NCLDVs and eukaryotes in individual habitat types are drawn as edges. Those NCLDV phylotypes and eukaryotic ASVs that were present in ≥ 10% of all soil samples for each habitat type were included in our co-occurrence network analysis. Significant Spearman correlation coefficients (*ρ* ≥ 0.60, *P* < 0.05) for NCLDVs-eukaryotes pairs were used as a cutoff. Experiment-verified and in silico horizontal gene transfer-based predicted virus–host associations reported in previous studies [4, 17] are shown in red and blue respectively (for details, please see Supplementary Table S9), whereas unknown associations are shown in grey. **b**–**e** Pearson correlations of the relative abundances between soil NCLDVs and algae, animals, fungi, and protozoa. Relative abundances were normalized by *z*-score

Using co-occurrence network analysis, we identified a total of 34, 42, 23, 12, and 36 NCLDV–eukaryote species pairs that showed strong linkages in farmland, forest, grassland, Gobi desert, and mine wasteland, respectively (Fig. 7a, Supplementary Fig. S9 and Supplementary Table S6). Only half of the phylotypes (14 out of 29) involved in these pairs could be assigned to a specific NCLDV family as *Asfarviridae*, *Iridoviridae*, *Mimiviridae*, *Pithoviridae*, and *Poxviridae*. Among the 136 distinct eukaryotic ASVs in the pairs, the majority were from fungi (55), followed by those from protozoa (49) and algae (26). There were significant positive correlations between the relative abundances of NCLDVs and their potential hosts (i.e., algae, animals, fungi, and protozoa) detected in the co-occurrence network (Fig. 7b–e). Similar patterns were obtained when an additional co-occurrence network analysis was performed to explore the associations between those 14 NCLDV phylotypes shared by at least four habitat types and eukaryotic ASVs (Supplementary Fig. S10). Of the 29 identified pairs, 18, 5, and 5 were NCLDV–fungi, NCLDV–protozoa, and NCLDV–algae associations, respectively. Remarkably, 16 out of the identified 18 NCLDV–fungi species pairs belonged to NCLDV–*Ascomycota* associations.

### Characteristics of soil NCLDV genomes recovered from this study

**Fig. 8:**
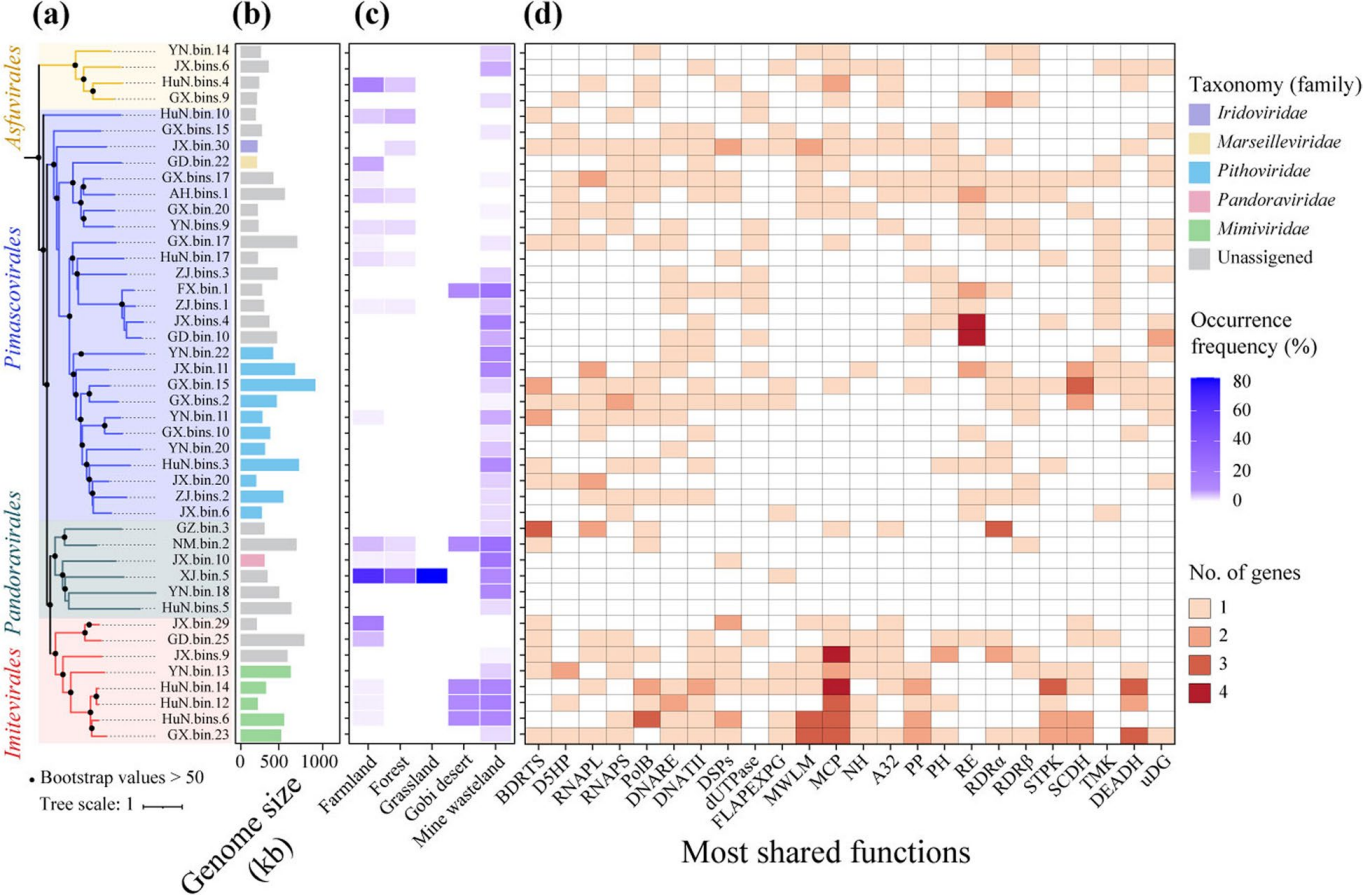
Analysis of 44 giant virus metagenome-assembled genomes (GVMAGs) recovered from our study. **a** Maximum-likelihood phylogenetic tree of the GVMAGs inferred from a concatenated protein alignment of seven core giant virus orthologous groups [14]. **b** Genome size and family-level taxonomic information of the GVMAGs. **c** Occurrence frequencies of individual GVMAGs in five different habitat types. **d** Comparison of most shared functions among the GVMAGs. Functions were selected among the annotations found in at least 10 genomes. ADPR, ADP-ribosylglycohydrolase; AlkB, NCLDV alkylated DNA repair protein; ATPDL, ATP-dependent DNA ligase; CtdP, ctd-like phosphatase; D5HP, D5-like helicase-primase; dMNPK, dNMP kinase; RNAPL, DNA-directed RNA polymerase subunit alpha; RNAPS, DNA-directed RNA polymerase subunit beta; SFII, DNA or RNA helicases of superfamily II; PolB, family B DNA polymerase; DNARE, DNA repair exonuclease; DNATII, DNA topoisomerase II; EL, Esterase lipase superfamily; FADTS, FAD-dependent thymidylate synthase; GT, glycosyltransferase; mRNACE, mRNA capping enzyme large subunit; NH, Nudix hydrolase; PP, Patatin phospholipase; RGTPase, Ras-like GTPase; RE, restriction-fold endonuclease; RDRα, ribonucleoside diphosphate reductase-α; RDRβ, ribonucleoside diphosphate reductase-β; STTPK, Serine/Threonine or Tyrosine-protein kinase; STPK, Serine/Threonine protein kinase; SCDH, short chain dehydrogenase; TMK, Thymidine kinase; DEADH, DEAD/SNF2-like helicases; uDG, uracil-DNA glycosylase; XRNE, XRN 5′-3′ exonuclease

To further explore the potential functions of soil NCLDVs, we recovered a total of 44 medium- to high-quality GVMAGs from our metagenomic data, which had a genome size ranging from 202 to 994 kb (Fig. 8, Supplementary Fig. S11 and Supplementary Table S7). Among the 44 GVMAGs, 36, 5, and 3 were recovered from mine wasteland, farmland, and forest, respectively (Supplementary Table S7). All these GVMAGs could be classified at the order level, with 4, 8, 6, and 26 being classified to *Asfuvirales*, *Imitervirales*, *Pandoravirales*, and *Pimascovirales*, respectively (Fig. 8a). However, only 19 of them could be further assigned to 5 known families (Fig. 8b and Supplementary Table S7): *Iridoviridae* (1), *Marseilleviridae* (1), *Mimiviridae* (5), *Pandoraviridae* (1), and *Pithoviridae* (11). The distributions of these GCMAGs were also heterogeneous among different habitat types (Fig. 8c), being consistent with the distribution patterns inferred from the *polB* phylotypes (Supplementary Table S4). For instance, the GVMAGs of *Mimiviridae* were detectable in farmland, Gobi desert, and mine wasteland, whereas those affiliated with *Pithoviridae* were detected mainly in mine wasteland.

While a majority (62% average) of the genes encoded by the 44 GVMAGs had no matches in the KEGG, NCVOG, and Pfam databases, a total of 38 known core genes were identified by comparing highly shared functions, including DNA topoisomerase II and *polB* (Fig. 8d and Supplementary Table S8). However, three presumably conserved NCLDV genes (i.e., MCP, packaging ATPase, and poxvirus late transcription factor VLTF3) [14] were absent from all of the recovered *Pithoviridae* genomes. Remarkably, 12 GVMAGs encoded a total of 20 glycoside hydrolase (GH) genes that spanned 13 GH gene families with capacities for polysaccharide degradation (e.g., cellulose, chitin, glucan, and pectate; Supplementary Table S8).

**Fig. 9:**
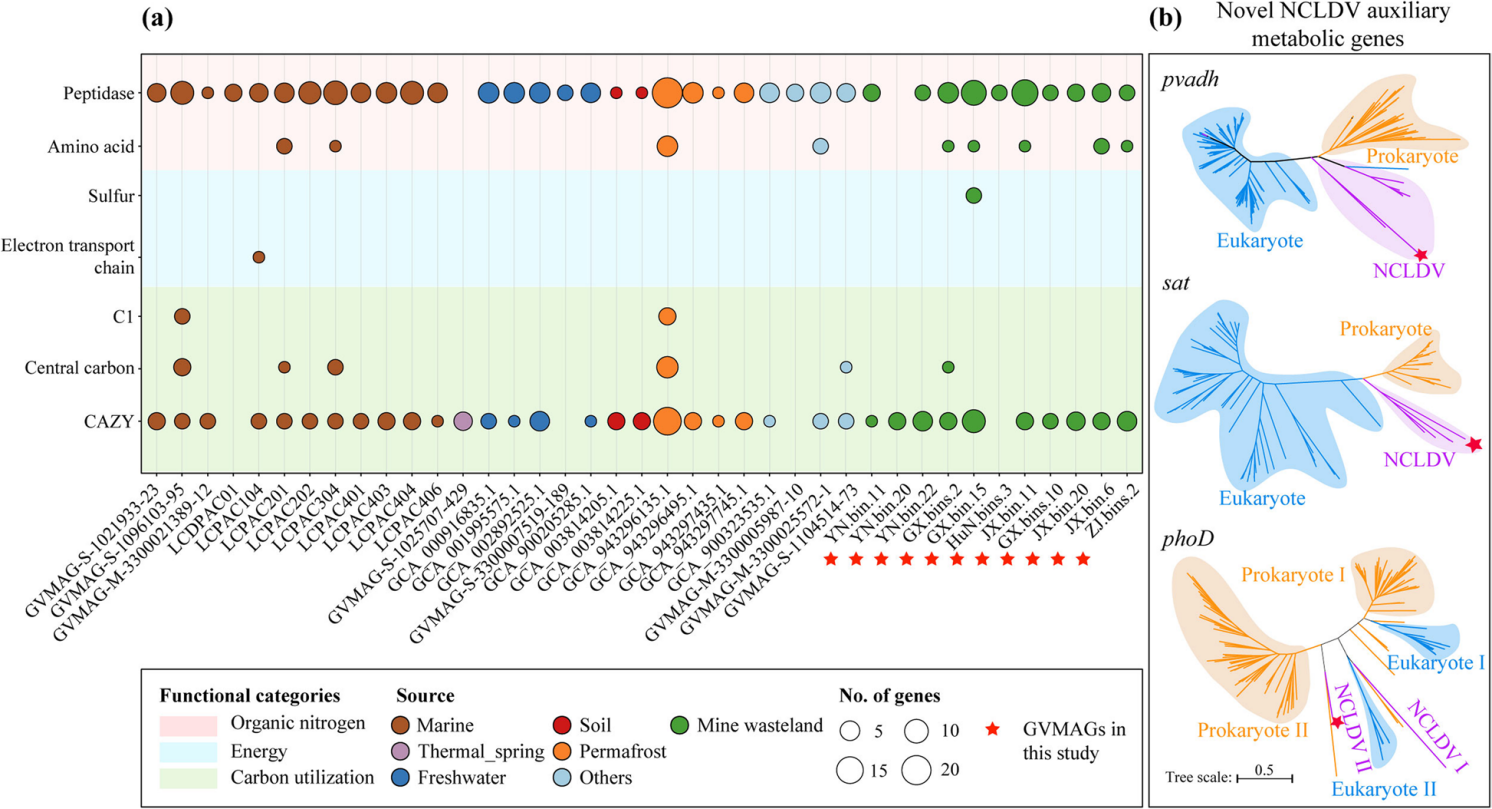
Functional analysis of *Pithoviridae-like* genomes. **a** Comparison of auxiliary metabolic functions between newly obtained and public reference *Pithoviridae* genomes [14, 19]. The 11 *Pithoviridae*-like genomes recovered in the study were marked with red stars (for details, please see Supplementary Tables S7 and S8). CAZY, carbohydrate-active enzymes. **b** Phylogenetic reconstruction of NCLDV genes likely involved in carbon, sulfur, and phosphorous metabolism. Asterisk denoted NCLDV sequences from the newly recovered *Pithoviridae*-like genomes. All the nodes were supported by > 75% bootstrap values, although they were not provided for better visual clarity. *pvadf*, polyvinyl alcohol dehydrogenase gene; *sat*, sulfate adenylyltransferase gene; *phoD*, alkaline phosphatase D gene

Through functional analysis of 11 newly recovered and 27 public reference *Pithoviridae*-like genomes, we found that certain genes involved in carbon and nitrogen cycling were commonly detected in *Pithoviridae* (Fig. 9a). However, similar to other NCLDV genomes, these *Pithoviridae*-like genomes showed a patchy distribution of auxiliary metabolic genes (AMGs; Fig. 9a and Supplementary Table S8). Some genes have not been reported previously, such as those encoding polyvinyl alcohol dehydrogenase (*pvadh*), sulfate adenylyltransferase (*sat*), and alkaline phosphatase D (*phoD*; Fig. 9b). Note that these genes were not only encoded by the *Pithoviridae* genomes but also identified in public *Pandoraviridae* (*pvadh*), *Marseilleviridae* and *Mimiviridae* genomes (*sat*). Phylogenetic analysis showed that these AMGs were likely acquired by giant viruses through horizontal gene transfer from eukaryotes or prokaryotes (Fig. 9b).

## Discussion

Using metagenomic sequencing, we characterized the broad diversity and wide distribution of soil giant virus phylotypes and compared them with those from marine environments. *Mimiviridae* is the major contributor of marine NCLDVs all over the world, as revealed by several metagenome-based studies [11, 13, 17]. In the first study addressing soil NCLDVs with metagenomics, Schulz et al. identified a high proportion of MCP genes positioned within *Mimiviridae* from forest soil samples [12]. High-throughput isolation of giant viruses also revealed that various mimiviruses can be isolated from diverse terrestrial environments [2, 3, 6]. In accordance with the previous studies, our results showed that *polB* phylotypes of *Mimiviridae* were present in all five habitat types (Figs. 1b and 4). Despite its great diversity, *Mimiviridae* is not the only major contributor of soil NCLDVs. The number of *polB* phylotypes assigned to *Pithoviridae* recovered in our study was higher than that of *Mimiviridae* (Fig. 1b and Supplementary Table S4). Moreover, *Pithoviridae* was also detected in all five habitat types, and even dominated in mine wasteland (Fig. 3e). Pithoviruses have been less explored before, as evidenced by the few isolates [6] and assembled genomes from marine or soil samples [13, 14]. However, a recent metagenomics study showed that *Pithoviridae* dominated the NCLDV communities in permafrost [19]. Nevertheless, our results revealed the hidden diversity of pithoviruses, which could be possibly more diverse and common than mimiviruses in soil.

The high diversity and abundance of *Pithoviridae* observed in the mine wasteland could highlight the enrichment of this viral family in such an environment. Moreover, of the five habitat types, mine wasteland harbored the most NCLDV phylotypes, where as many as 83% of phylotypes were habitat-specific (Fig. 2b). These suggested that mine wasteland represents a “hotspot” of unique NCLDV phylotypes. Mine wasteland is generally known as an acidic, oligotrophic, and toxic environment, given that the sulfur-bearing metal-rich mine wastes on it are readily oxidized and solubilized by iron- and sulfur-oxidizing microorganisms [47]. Such edaphic uniqueness likely contributed to the occurrence of a high number of unique NCLDV phylotypes in this habitat type. For instance, among all soil samples collected in this study, only a proportion of those from mine wasteland had a pH value < 4, wherein 63 distinct phylotypes belonging to six NCLDV families were recovered (Supplementary Fig. S12). To our knowledge, only two partial genomes of NCLDVs have been reported in acidic environments (i.e., acid mine drainage) previously [48].

The heterogeneous distribution of soil giant viruses in different habitat types (Fig. 2 and Supplementary Table S4) could be attributed to climatic conditions, soil physicochemical characteristics, geographical barriers, and host distribution [17, 49]. A recent metagenomic study revealed that historical precipitation affected soil phage life cycle, host diversity, and host-phage interaction in grassland [50]. Changes in precipitation led to a shift in soil moisture, which was the main driving factor for viral abundance in grassland; and soil tended to have more diverse viruses under the condition of high moisture content [51]. In consistence with the previous findings, we found that MAP was the most common important environmental factor affecting β diversity of soil NCLDVs in all five habitat types and also affected their α diversity in three habitat types (Figs. 5 and 6).

Soil pH was the common environmental factor influencing β diversities of soil NCLDVs in farmland, forest, grassland, and mine wasteland (Fig. 6). Likewise, a previous study reported that the major environmental driver of phage community structure in Antarctic soil was pH [52]. Of note, it has been even validated by experiments that pH affected the adsorption of phages to soils, thereby profoundly influencing the diversity of soil phages and their interaction with hosts [53]. Other edaphic factors like Ex-Ca were also among the determinants of β diversities of soil NCLDVs (Fig. 6), and its effects on soil phage survival and community composition have been reported previously as well [52, 53].

Note that both α and β diversities of soil giant viruses were unlikely affected directly by the abovementioned abiotic factors, but rather most likely by their hosts [17, 54]. In fact, strong direct influences of prokaryotes on their phage communities have been frequently observed in previous studies [55, 56]. In our study, soil eukaryotic community composition alone was able to explain > 20% of the total variance of β diversities of soil NCLDVs in individual habitat types, being greater than the sum of those explained by climatic, geographical, and physicochemical factors considered in this study (Fig. 6a–e). Additionally, even after controlling for the effect of geographic distance, soil eukaryotic community composition was the most influential environmental factor that was significantly correlated with soil NCLDV community composition (*P* < 0.01 in all five habitat types, Fig. 6f). These results are reasonable, given that soil eukaryotes, in theory, are potential hosts of soil giant viruses [1, 4].

Co-occurrence network analysis of NCLDV *polB* genes and eukaryotic 18S rRNA gene has been employed to explore potential NCLDV-host pairings in the ocean, revealing that particular microeukaryotes (e.g., *Alveolata*, *Opisthokonta*, *Rhizaria*, and *Stramenopiles*) are potential hosts of NCLDVs [4, 17, 18, 57]. Using the same approach, our study detected tight linkages between soil NCLDVs and eukaryotic species from *Amoebozoa*, *Rhizaria* (mainly *Cercozoa*), *Chloroplastida* (*Chlorophyta*), *Stramenopiles* (*Ochrophyta*) and fungi (*Ascomycota*) (Fig. 7, Supplementary Fig. S9 and Supplementary Table S6). Specifically, two NCLDV families (*Asfarviridae* and *Mimiviridae*) were found to be strongly correlated with *Amoebozoa*, which was in accordance with previous reports that many members of these two families are amoeba-infecting viruses (Supplementary Table S9) [6, 17]. Although no cercozoan hosts of NCLDVs have been isolated, recent metagenomics and metatranscriptomics studies provided evidence of possible *Phycodnaviridae-* and *Mimiviridae*-*Cercozoa* pairings [58, 59]. Besides *Phycodnaviridae* and *Mimiviridae*, our results revealed two more NCLDV families (i.e., *Asfarviridae* and *Iridoviridae*) having tight linkages with members of *Cercozoa*. Both our results and previous co-occurrence analysis indicated that algae could be potential hosts of *Pithoviridae*, with one associated with *Cryptomonadaceae* [19] and another with *Chrysophyceae* (this study). Although fungi have not yet been reported as experimentally verified hosts of NCLDVs, they are considered potential NCLDV hosts based largely on several lines of evidence regarding the genes or genomic fragments shared by them and NCLDVs [13, 60–62].

While the putative NCLDV–host pairings recorded in this study remain to be experimentally verified, they indicate that NCLDVs probably have an innegligible effect on soil biogeochemistry via affecting their hosts. Some NCLDVs have been reported to be able to result in complete host cell lysis [6]. Of note, algae, fungi and protozoa harboring most of the putative hosts identified in this study are among the important players in soil biogeochemical cycling. For instance, soil algae have been estimated to take up about 3.6 Pg C annually (equal to ca. 6% of the net primary production of terrestrial vegetation) [63], and soil fungi are known for their ability to decompose organic matter [64].

The recovery of 44 GVMAGs in our study not only considerably expanded the genome diversity of soil NCLDVs but also helped to reveal new insights into their genetic features (Fig. 8). About 60% of the 44 GVMAGs were unassigned at the family level, which constituted several new clades within the *Asfuvirales*, *Imitervirales*, *Pandoravirales* and *Pimascovirales* orders (Supplementary Fig. S11). Meanwhile, the addition of 11 *Pithoviridae-*like genomes to the currently available 27 public *Pithoviridae* genomes (only 2 from forest soil [14] and 4 from permafrost [19]; Fig. 9) led to a 41% increase in the number of *Pithoviridae* genomes. Remarkably, one *Pithoviridae* genome recovered in this study (GX.bin.15; 994 kb; Supplementary Table 7) was larger than those (ca. 550 kb) of the isolated *Pithoviridae* [14], while the other *Pithoviridae-*like genomes of this study had a size (ca. 500 kb) similar to those of the *Pithoviridae* isolates.

Previous studies have reported that giant viruses can encode AMGs to augment and/or modulate the metabolic capabilities of the host cell [4, 11, 13]. For instance, some genes involved in nutrient acquisition (e.g., phosphate permease) and light-driven energy generation (e.g., rhodopsin) were commonly found in giant virus genomes from aquatic environments [4, 11, 13]. However, such AMGs were seldom found in the GVMAGs recovered in this study (Supplementary Table S8), indicating that certain AMGs could be aquatic-specific. Additionally, we were not able to find any AMG that was ubiquitous in a given lineage of NCLDVs. Overall, our analysis revealed a patchwork of AMGs of soil giant viruses amidst a larger reservoir of genes of unknown functions (Supplementary Table S8), which was consistent with the pattern observed for giant viruses in permafrost [19].

Among the 13 GH gene families encoded by our soil GVMAGs (Supplementary Table S8), GH18 chitinase genes have been found in a few giant viruses (e.g., *Chlorovirus*, *Satyrvirus*, and *Fadolivirus*), which led to the hypothesis that these viruses possess chitinase genes to degrade the chitin polymers present in host cell wall upon infection [65]. However, such a hypothesis seemed not to be fully supported by the fact that the cell wall of certain infected hosts was not composed of chitin [66]. Interestingly, there was evidence that the pandoravirus MVP2 evolved from an inactivated GH16 family glycoside hydrolase [67]. Except for GH18 and GH16, the other 11 GH gene families have been less explored in giant viruses [4, 11, 13]. The occurrence of a set of diverse and previously underexplored GH gene families in our GVMAGs indicates that giant viruses may be closely involved in soil carbon cycling. In agreement with this notion, an AMG (i.e., *pvadh*) encoding polyvinyl alcohol dehydrogenase was detected in one *Pithoviridae* genome recovered in this study (Fig. 9b). Moreover, the detection of two additional novel AMGs (*sat* and *phoD*) in our *Pithoviridae* genomes revealed for the first time that pithoviruses had the potential to participate in soil sulfur and phosphorus cycling.

Although the purpose of this study was not to make a comparison between soil and oceanic giant viruses, we have noticed that the average number of distinct NCLDV *polB* genes recovered from our soil samples (approximately 1.6 genes per sample) was much lower than that (approximately 24 genes per sample) recorded by Endo and colleagues using almost the same shotgun metagenomics (at a sequencing depth of about 30 G per sample) for the < 3 µm size fractions of the global seawater samples [17]. One possible reason for the much lower number of NCLDV *polB* genes recovered in our study was that the higher complexity of microbial communities in soils than in seawater [68] hindered considerably the recovery of the marker gene for our metagenomes. Meanwhile, we have observed that the number of NCLDV *polB* genes detected in our soil samples was positively correlated to sequencing depth (Supplementary Fig. S13). Such a pattern indicates that deeper sequencing likely helps to capture more diversity of soil NCLDV *polB* genes. However, it should be also noted that using a microfluidic-based mini-metagenomics that combines the advantages of shotgun and single-cell metagenomic analyses [69], Schulz and colleagues were able to recover as many as 15 GVMAGs from 4 forest soil samples at a sequencing depth of about 0.5 G per sample [12]. Taken together, due to the relatively low sequencing depth and the lack of an approach for de-convolving NCLDVs’ DNA from complex soil samples, the typical shotgun metagenomics used in this study had limited our ability to capture a higher phylogenetic or genomic diversity of soil giant viruses. Therefore, more studies utilizing ultra-deep metagenomic sequencing, microfluidic-based mini-metagenomics, and/or metaviromics are needed to reach a more comprehensive understanding of the phylogenetic and genomic diversity of giant viruses in our soil samples.

## Conclusions

In summary, the analysis of NCLDV *polB* genes recovered in this study uncovered that the phylotype diversity of giant viruses in soil environments was much higher than currently recognized and that the distribution of these phylotypes was heterogeneous among habitat types. Soil eukaryotes were identified as the most important driver of beta-diversity of giant viral communities across habitat types. The co-occurrence analysis of NCLDV phylotypes and eukaryotes linked giant viruses to protozoa, fungi, and algae. Functional analysis of 44 newly recovered giant virus genomes revealed novel auxiliary metabolic genes related to carbon, sulfur, and phosphorus cycling. These findings extend our knowledge of the habitat preferences, ecological drivers, potential hosts, and auxiliary metabolism of soil giant viruses.

### Supplementary Information

Supplementary Material 1: Supplementary Table S1. Detailed information of the sampling sites of this study and their corresponding environmental factors. Supplementary Table S2. Information on metagenomics data used in this study. Supplementary Table S3. PolB sequences used to benchmark our phylogenetic mapping approach (as per Kazlauskas et al. [31]). Supplementary Table S4. Selected detailed information on the soil NCLDV phylotypes identified in this study. Supplementary Table S5. Taxonomic affiliations of marine NCLDV phylotypes identified in a previous study (Endo et al. [17]). Supplementary Table S6. Detailed information of soil NCLDV-eukaryote pairs in the co-occurrence networks shown in Supplementary Figure S9. Supplementary Table S7. Detailed information on giant virus metagenome-assembled genomes recovered in this study. Supplementary Table S8. Functional annotation of giant virus metagenome-assembled genomes recovered in this study. Supplementary Table S9. Reported NCLDVs-eukaryotes relationships in the references.

Supplementary Material 2: Supplementary Figure S1. Length distributions of NCLDV polB sequences recovered from this study (a) and from Tara Oceans (b). The pie charts shown in the insets denote the percentages of polB sequences ≥ 1 kb and < 1 kb. The polB sequences from Tara Oceans were obtained from Endo et al. [1]. Supplementary Figure S2. Sample-size dependence of the observed NCLDV phylotypes in this study. Sample-based rarefaction curves showing accumulated richness of NCLDV polB genes detected in individual habitat types. Supplementary Figure S3. Relationships between environmental ranges of individual NCLDV phylotypes and the numbers of sampling sites where they occurred (a) or the total abundances of individual phylotypes in all sampling sites (b). Each dot in each panel denotes a NCLDV phylotype. The color intensity of a given dot represents the number of habitat types where that NCLDV phylotype could be recovered. The solid red lines represent the linear regression models with statistically significant Pearson coefficients (*P* < 0.001). The total abundance of each phylotype and environmental range in (b) are normalized by logarithm. Supplementary Figure S4. Correlations between average abundances of the NCLDV phylotypes belonging to individual families and the numbers of sampling sites where the corresponding phylotypes could be detected. Each dot in each panel denotes a NCLDV phylotype. The color intensity of a given dot represents the number of habitats where that NCLDV phylotype could be recovered. The solid red lines represent the linear regression with statistically significant Pearson coefficients (*P* < 0.01). The phylotypes affiliated with Phycodnaviridae and Prasinoviridae were excluded for analysis due to the limited number of sampling sites (*n* < 3) where these phylotypes could be detected. Supplementary Figure S5. Variable clustering for assessment of the environmental variable redundancy. Environmental variables with Spearman r2 > 0.7 are excluded from subsequent analyses. LAT, latitude; ALT, altitude; MAP, mean annual 66 precipitation; EC, electrical conductivity; EX-Ca, exchangeable calcium; CEC, cation exchange capacity; TC, total carbon; TN, total N; TP, total P; TK, total K. Supplementary Figure S6. Relationships between selected environmental factors and NCLDV phylotype richness in individual habitat types. Colors of dots in each panel represent habitat types. Each dot represents one soil sample. The solid blue lines represent the linear regression with statistically significant Pearson coefficients. The solid red curves represent the polynomial fit determined on the basis of the corrected Akaike Information Criterion (AIC). Abbreviations are as those in Supplementary Figure S4. Supplementary Figure S7. Relative similarity of all samples in NCLDV community composition. (a) Non-metric multidimensional scaling (NMDS) ordination biplot showing the relative similarity of all samples. Samples are grouped and color-coded by habitat types. All groups are significantly different from each other as analyzed using Adonis (*P* = 0.001). (b) Results of multilevel pairwise comparison between habitat types. It was performed by pairwise.adonis from the package “pairwiseAdonis”. Supplementary Figure S8. The distance–decay relationships for soil NCLDV communities in individual habitat types. Pairwise NCLDV community dissimilarity (Bray–Curtis) significantly increases with pairwise geographic distance in the five habitat types: farmland (a), forest (b), grassland (c), Gobi desert (d) and mine wasteland (e). Supplementary Figure S9. The co-occurrence networks of NCLDVs–eukaryotes in different habitat types. Those NCLDV phylotypes and eukaryotic amplicon sequence variants (ASVs) that were present in ≥ 10% of all soil samples for each habitat type were included in our co-occurrence network analysis. Triangles represent eukaryotic ASVs and circles represent NCLDV phylotypes. The sizes of triangles and circles are proportional to the number of connections. Significant Spearman correlation coefficients (ρ ≥ 0.60, *P* < 0.05) for NCLDVs-eukaryotes pairs are drawn as edges. Supplementary Figure S10. The co-occurrence networks of the 14 ubiquitous NCLDVs across four or five habitat types and eukaryotic ASVs. Triangles represent eukaryotic ASVs and circles represent NCLDV phylotypes. Significant Spearman correlation coefficients (ρ ≥ 0.60, *P* < 0.05) for NCLDV-eukaryote pairs are drawn as edges. Supplementary Figure S11. The maximum-likelihood phylogenetic tree of giant virus metagenome-assembled genomes (GVMAGs) reconstructed in this study and available in public databases [2]. The tree was built from a concatenated protein alignment of seven marker genes (SFII, RNAPL, PolB, TFIIB, TopoII, A32 and VLTF3) using the model of LG + I + F + G4 and rooted at Poxviridae [2]. Tree branches are colored according to the order-level taxonomic assignment. The GVMAGs recovered from this study are labeled in red background. The outer strip is colored according to the family level taxonomic assignment. SFII, DEAD/SNF2-like helicase; RNAPL, DNA directed RNA polymerase alpha subunit; PolB, DNA polymerase family B; TFIIB, transcription initiation factor IIB; TopoII, DNA topoisomerase II; A32, Packaging ATPase; VLTF3, Poxvirus late transcription factor VLTF3. Supplementary Figure S12. The pH-relevant distribution profiles of the numbers of phylotypes belonging to individual NCLDV families. The color intensity of a given grid is proportionate to the number of NCLDV phylotypes belonging to a specific family that can be observed in a given pH range. Given that some phylotypes can occur in a wide range of soil pH, the sum of the numbers shown in the figure is greater than the total number of the NCLDV phylotypes identified in this study. Supplementary Figure S13. Relationships between and the number of NCLDV polB genes detected in individual samples and sequencing depth. Each dot in each panel represents one soil sample. The solid red lines represent the linear regression with statistically significant Pearson coefficients.

## Data Availability

The assembled *polB* sequences and assembled eukaryotic ASVs have been deposited in the European Nucleotide Archive (ENA) database under the accession numbers PRJEB53185. The giant virus metagenome-assembled genomes have been deposited in the ENA database under the accession number PRJEB74361. The code used for analysis in this study is freely available online (https://github.com/liangjlgit/NCLDV_code_R).

## Abbreviations

ADPR: ADP-ribosylglycohydrolase
AlkB: NCLDV alkylated DNA repair protein
ALT: Altitude
ATPDL: ATP-dependent DNA ligase
ASV: Amplicon sequence variant
CtdP: Ctd-like phosphatase
D5HP: D5-like helicase-primase
DEADH: DEAD/SNF2-like helicases
dMNPK: DNMP kinase
DNARE: DNA repair exonuclease
DNATII: DNA topoisomerase II
EL: Esterase lipase superfamily
FADTS: FAD-dependent thymidylate synthase
GT: Glycosyltransferase
GVMAG: Giant virus metagenome-assembled genome
LAT: Latitude
LON: Longitude
MAG: Metagenome-assembled genome
MAP: Mean annual precipitation
MAT: Mean annual temperature
MCP: Major capsid protein
mRNACE: MRNA capping enzyme large subunit
MSE: Mean square error
NCLDV: Nucleocytoplasmic large DNA virus
NH: Nudix hydrolase
NMDS: Non-metric multidimensional scaling
phoD: Alkaline phosphatase D
PolB: Family B DNA polymerase
PP: Patatin phospholipase
pvadh: Polyvinyl alcohol dehydrogenase
RDRα: Ribonucleoside diphosphate reductase-α
RDRβ: Ribonucleoside diphosphate reductase-β
RE: Restriction-fold endonuclease
RGTPase: Ras-like GTPase
RNAPL: DNA-directed RNA polymerase subunit alpha
RNAPS: DNA-directed RNA polymerase subunit beta
sat: Sulfate adenylyltransferase
SCDH: Short chain dehydrogenase
SEV: Standardized environmental variable
SFII: DNA or RNA helicases of superfamily II
STPK: Serine/threonine protein kinase
STTPK: Serine/threonine or tyrosine-protein kinase
TC: Total carbon
TK: Total K
TMK: Thymidine kinase
TN: Total N
TP: Total P
uDG: Uracil-DNA glycosylase
VPA: Variation partition analysis
XRNE: XRN 5′-3′ exonuclease
