# Supplementary material for: Unraveling the habitat preferences, ecological drivers, potential hosts, and auxiliary metabolism of soil giant viruses across China
Source: Microbiome. 2024 Jul 22;12:136. doi: 10.1186/s40168-024-01851-8 (PMC11265010; doi:10.1186/s40168-024-01851-8)
Supplement: Supplementary file 3 — Supplementary Material 2: Supplementary Figure S1. Length distributions of NCLDV polB sequences recovered from this study (a) and from Tara Oceans (b). The pie charts shown in the insets denote the percentages of polB sequences ≥ 1 kb and < 1 kb. The polB sequences from Tara Oceans were obtained from Endo et al. [1]. Supplementary Figure S2. Sample-size dependence of the observed NCLDV phylotypes in this study. Sample-based rarefaction curves showing accumulated richness of NCLDV polB genes detected in individual habitat types. Supplementary Figure S3. Relationships between environmental ranges of individual NCLDV phylotypes and the numbers of sampling sites where they occurred (a) or the total abundances of individual phylotypes in all sampling sites (b). Each dot in each panel denotes a NCLDV phylotype. The color intensity of a given dot represents the number of habitat types where that NCLDV phylotype could be recovered. The solid red lines represent the linear regression models with statistically significant Pearson coefficients (P < 0.001). The total abundance of each phylotype and environmental range in (b) are normalized by logarithm. Supplementary Figure S4. Correlations between average abundances of the NCLDV phylotypes belonging to individual families and the numbers of sampling sites where the corresponding phylotypes could be detected. Each dot in each panel denotes a NCLDV phylotype. The color intensity of a given dot represents the number of habitats where that NCLDV phylotype could be recovered. The solid red lines represent the linear regression with statistically significant Pearson coefficients (P < 0.01). The phylotypes affiliated with Phycodnaviridae and Prasinoviridae were excluded for analysis due to the limited number of sampling sites (n < 3) where these phylotypes could be detected. Supplementary Figure S5. Variable clustering for assessment of the environmental variable redundancy. Environmental variables with Spearman r2 > 0.7 are excluded [file 40168_2024_1851_MOESM2_ESM.pdf]

Supplementary figures for

Unravelling the habitat preferences, ecological drivers, potential hosts and auxiliary metabolism of soil giant viruses across China

Jie-Liang Liang[1,†], Shi-wei Feng[1,†], Pu Jia[1], Jing-li Lu[1], Xinzhu Yi[1], Shao-ming Gao[2], Zhuo-hui Wu[1], Bin Liao[2], Wen-sheng Shu[1] & Jin-tian Li[1,*]

[1]Institute of Ecological Science, Guangzhou Key Laboratory of Subtropical Biodiversity and Biomonitoring, Guangdong Provincial Key Laboratory of Biotechnology for Plant Development, School of Life Sciences, South China Normal University, Guangzhou 510631, PR China

[2]School of Life Sciences, Sun Yat-sen University, Guangzhou 510275, PR China

Jie-Liang Liang: liangjl@m.scnu.edu.cn

Shi-wei Feng: 2018022499@m.scnu.edu.cn

Pu Jia: pjia@m.scnu.edu.cn

Jing-li Lu: 2018010179@m.scnu.edu.cn

Xinzhu Yi: yizinzhu@m.scnu.edu.cn

Shao-ming Gao: gaoshaom@mail2.sysu.edu.cn

Zhuo-hui Wu: 2019022501@m.scnu.edu.cn

Bin Liao: liaobin2005@126.com

Wen-sheng Shu: shuwensheng@m.scnu.edu.cn

Jin-tian Li: lijintian@m.scnu.edu.cn

†These two authors contributed equally to this work.

*Corresponding author:

School of Life Sciences, South China Normal University, Guangzhou 510631, PR China

E-mail: lijintian@m.scnu.edu.cn

Tel.: +86 20 85211850; Fax: +86 20 85211850

Running title: Biogeography and ecology of soil giant viruses

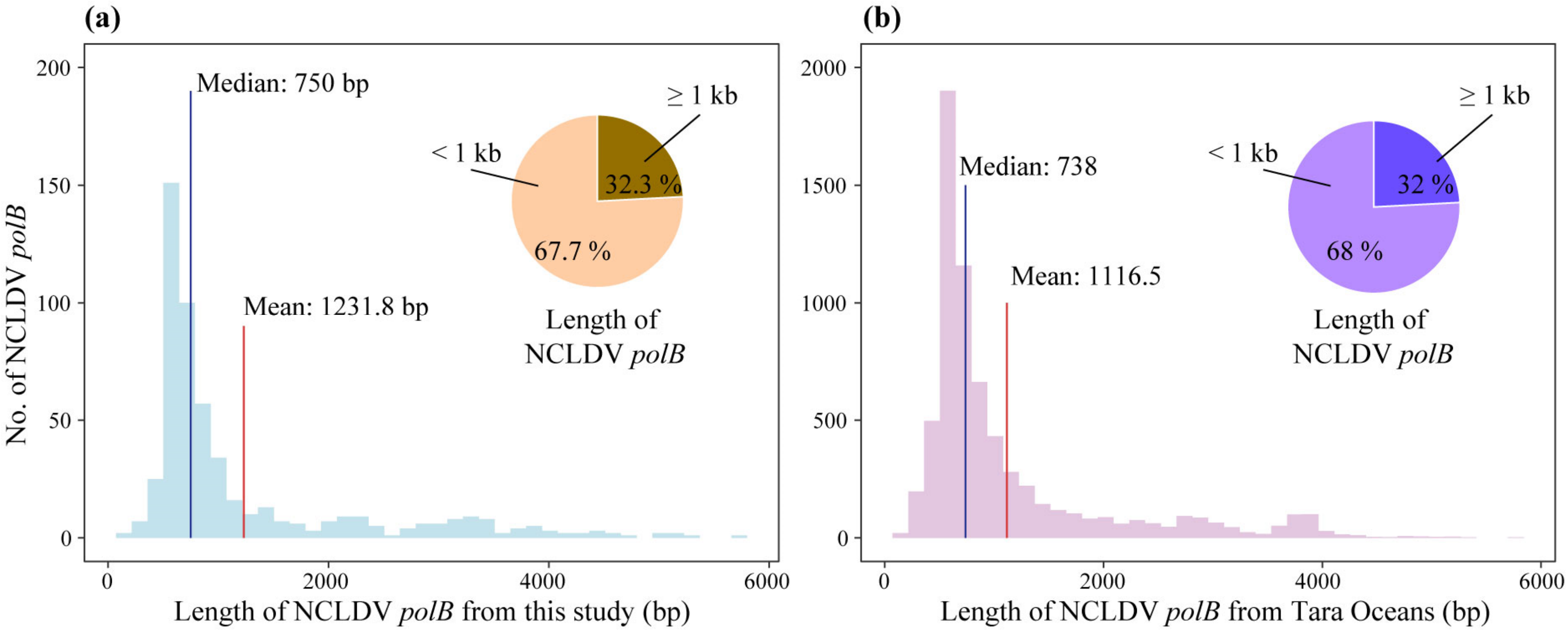

Supplementary Figure 1. Length distributions of NCLDV *polB* sequences recovered from this study (a) and from Tara Oceans (b). The pie charts shown in the insets denote the percentages of *polB* sequences ≥ 1 kb and < 1 kb. The *polB* sequences from Tara Oceans were obtained from Endo *et al*. [1].

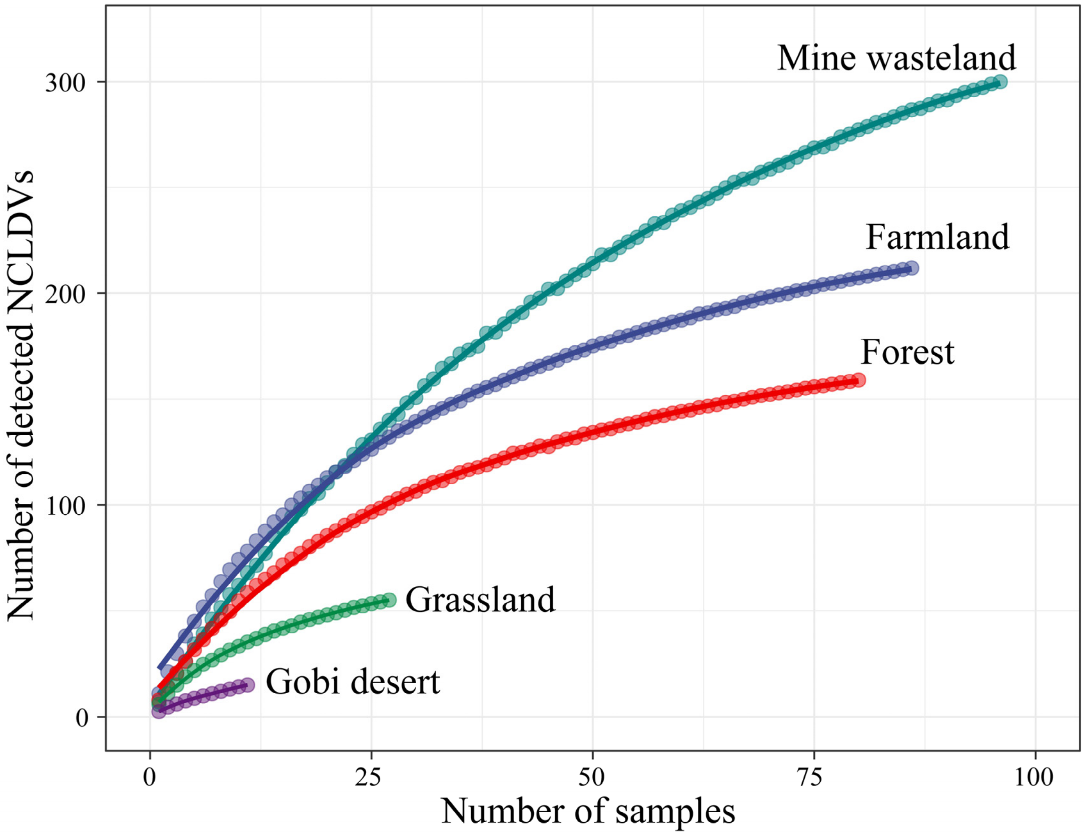

Supplementary Figure 2. Sample-size dependence of the observed NCLDV phylotypes in this study. Sample-based rarefaction curves showing accumulated richness of NCLDV *polB* genes detected in individual habitat types.

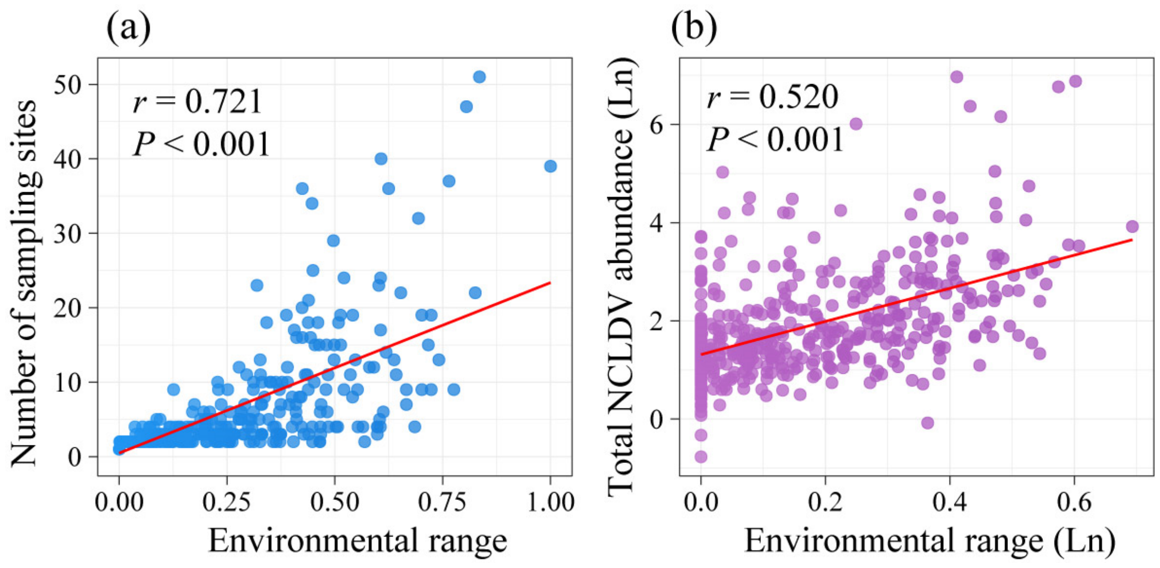

Supplementary Figure 3. Relationships between environmental ranges of individual NCLDV phylotypes and the numbers of sampling sites where they occurred (a) or the total abundances of individual phylotypes in all sampling sites (b). Each dot in each panel denotes a NCLDV phylotype. The color intensity of a given dot represents the number of habitat types where that NCLDV phylotype could be recovered. The solid red lines represent the linear regression models with statistically significant Pearson coefficients ($P < 0.001$). The total abundance of each phylotype and environmental range in **(b)** are normalized by logarithm.

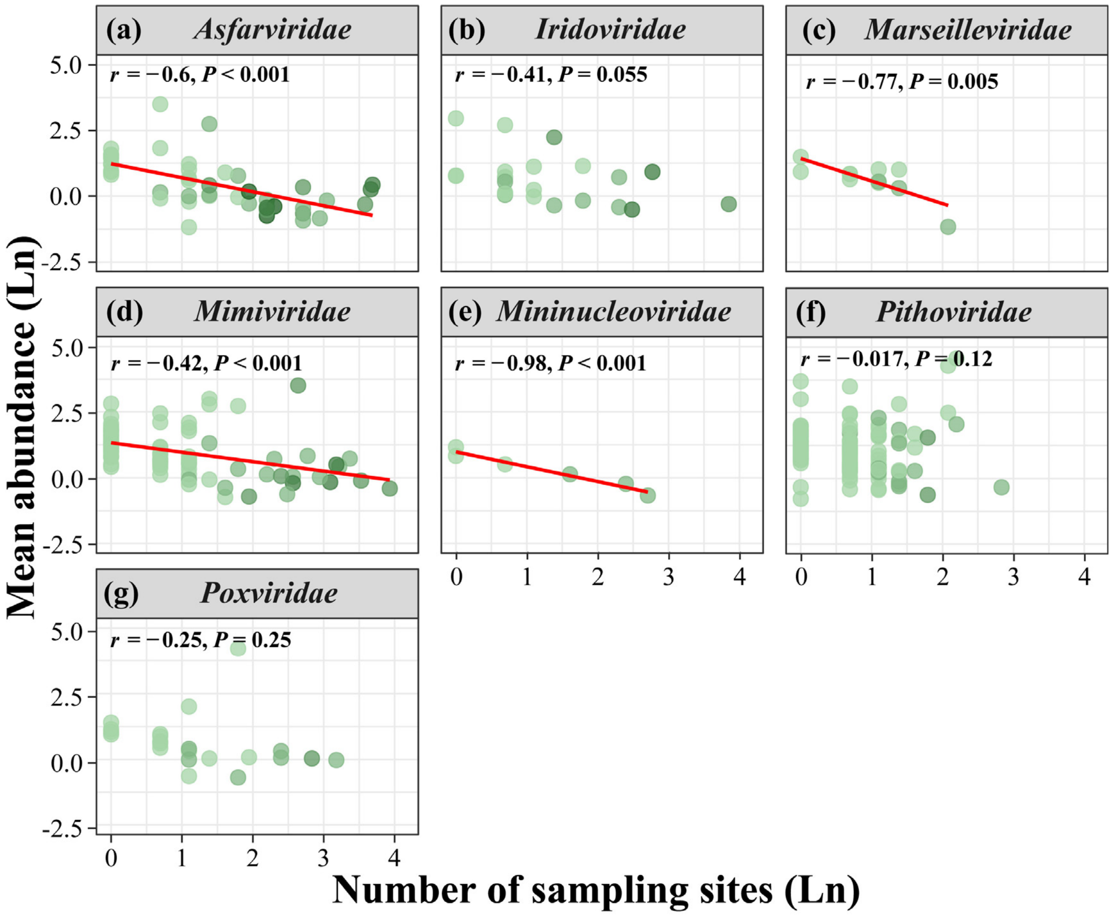

Supplementary Figure 4. Correlations between average abundances of the NCLDV phylotypes belonging to individual families and the numbers of sampling sites where the corresponding phylotypes could be detected. Each dot in each panel denotes a NCLDV phylotype. The color intensity of a given dot represents the number of habitats where that NCLDV phylotype could be recovered. The solid red lines represent the linear regression with statistically significant Pearson coefficients ($P < 0.01$). The phylotypes affiliated with *Phycodnaviridae* and *Prasinoviridae* were excluded for analysis due to the limited number of sampling sites ($n < 3$) where these phylotypes could be detected.

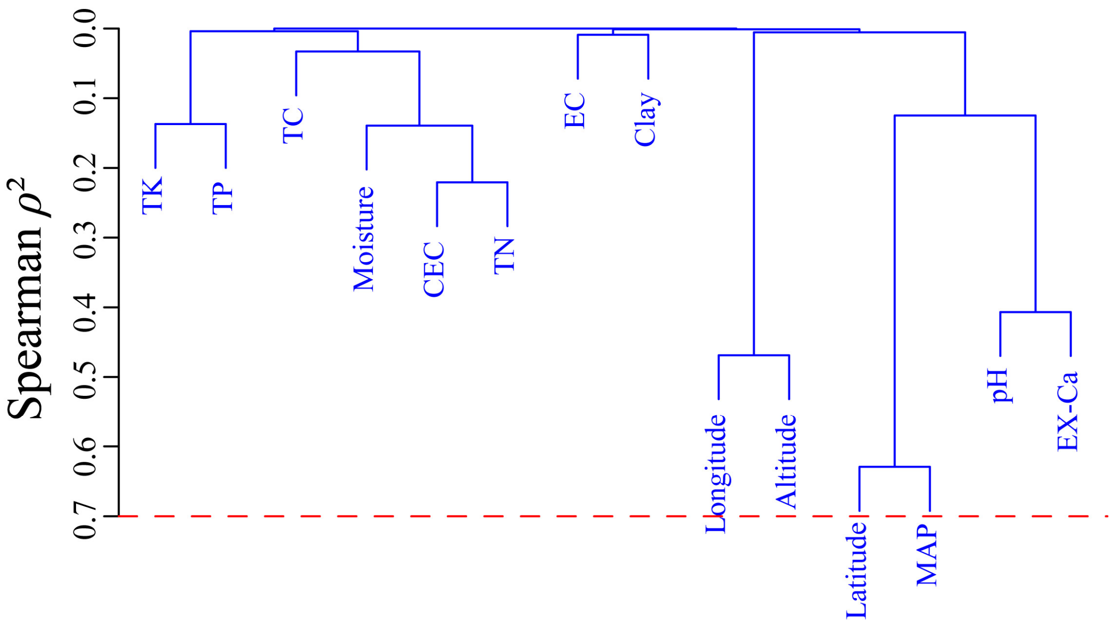

Supplementary Figure 5. Variable clustering for assessment of the environmental variable redundancy. Environmental variables with Spearman $r^2 > 0.7$ are excluded from subsequent analyses. LAT, latitude; ALT, altitude; MAP, mean annual precipitation; EC, electrical conductivity; EX-Ca, exchangeable calcium; CEC, cation exchange capacity; TC, total carbon; TN, total N; TP, total P; TK, total K.

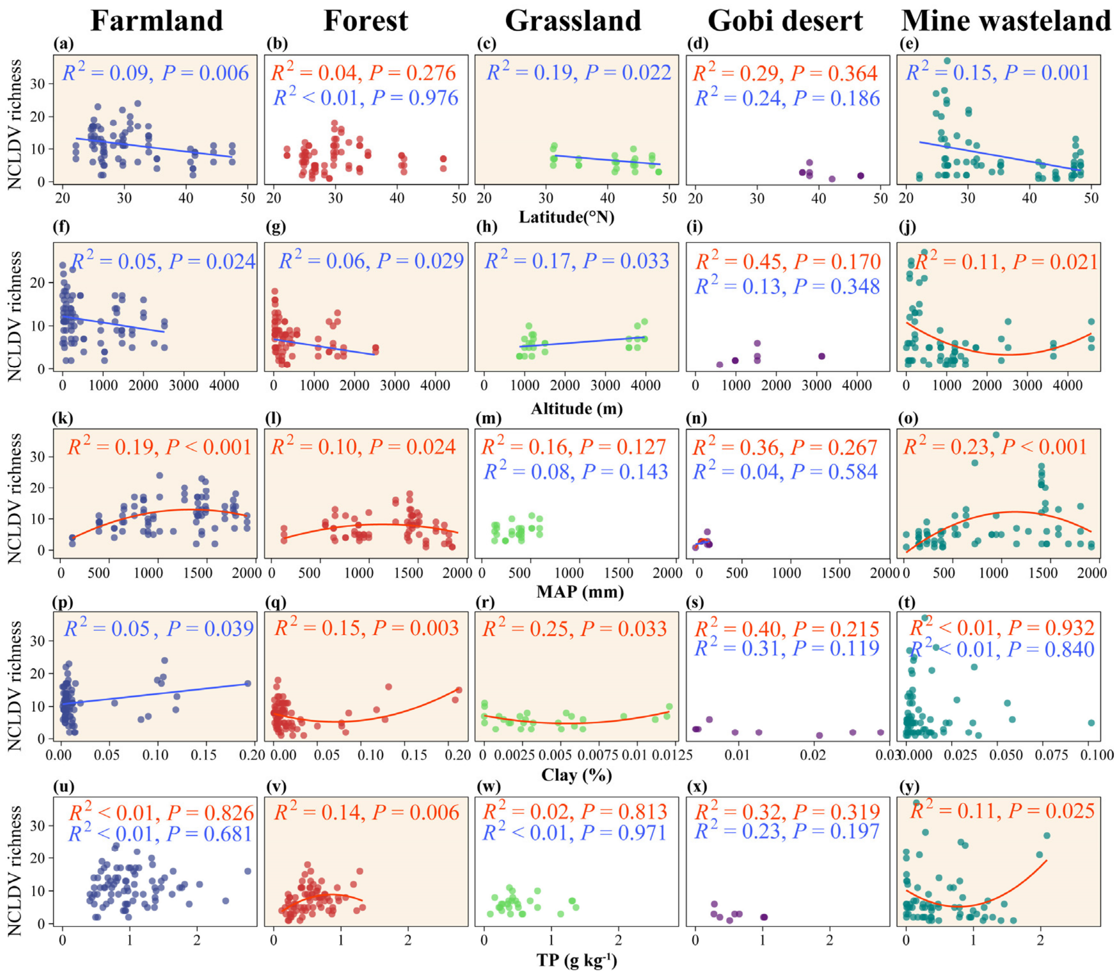

Supplementary Figure 6. Relationships between selected environmental factors and NCLDV phylotype richness in individual habitat types. Colors of dots in each panel represent habitat types. Each dot represents one soil sample. The solid blue lines represent the linear regression with statistically significant Pearson coefficients. The solid red curves represent the polynomial fit determined on the basis of the corrected Akaike Information Criterion (AIC). Abbreviations are as those in Supplementary Figure 4.

(a)

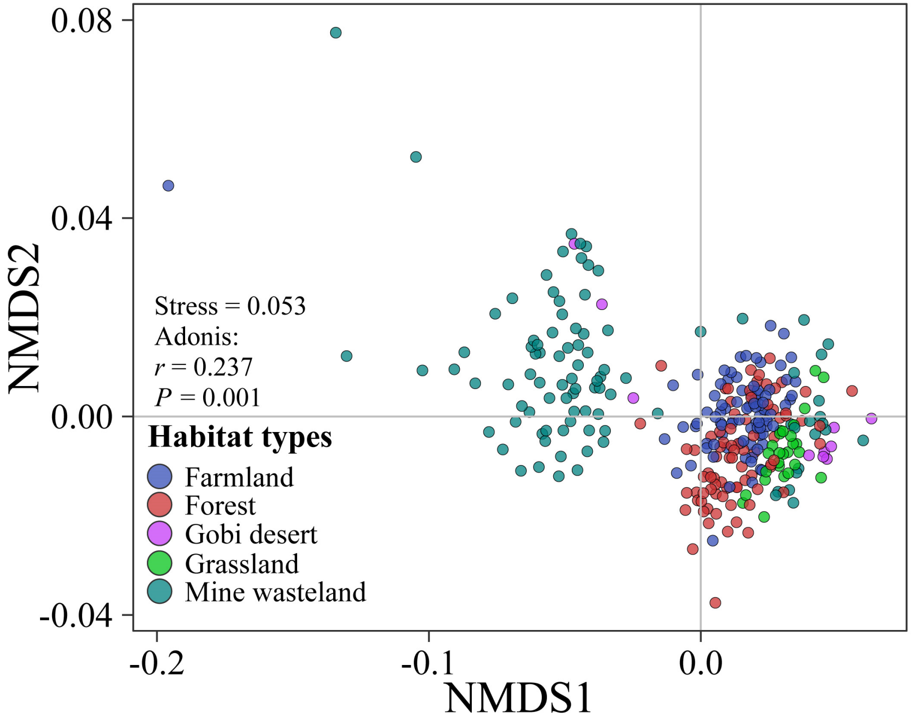

(b) Multilevel pairwise comparison

**Pairs**	***R*²**	***P*.value**	***P*.adjusted**
Forest *vs* Grassland	0.038	0.001	0.001
Forest *vs* Mine wasteland	0.025	0.001	0.001
Forest *vs* Farmland	0.021	0.001	0.001
Forest *vs* Gobi desert	0.048	0.001	0.001
Grassland *vs* Mine wasteland	0.033	0.001	0.001
Grassland *vs* Farmland	0.041	0.001	0.001
Grassland *vs* Gobi desert	0.117	0.001	0.001
Mine wasteland *vs* Farmland	0.033	0.001	0.001
Mine wasteland *vs* Gobi desert	0.034	0.001	0.001
Farmland *vs* Gobi desert	0.051	0.001	0.001

Supplementary Figure 7. Relative similarity of all samples in NCLDV community composition. (a) Non-metric multidimensional scaling (NMDS) ordination biplot showing the relative similarity of all samples. Samples are grouped and color-coded by habitat types. All groups are significantly different from each other as analyzed using Adonis (P = 0.001). (b) Results of multilevel pairwise comparison between habitat types. It was performed by pairwise.adonis from the package “pairwiseAdonis”.

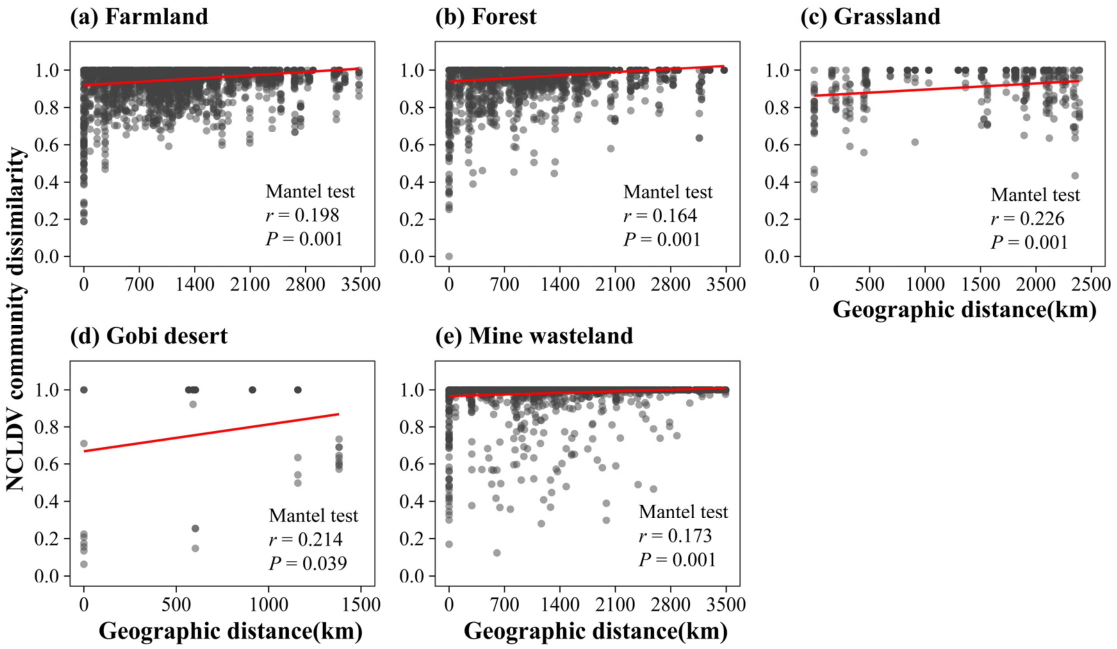

Supplementary Figure 8. The distance–decay relationships for soil NCLDV communities in individual habitat types. Pairwise NCLDV community dissimilarity (Bray-Curtis) significantly increases with pairwise geographic distance in the five habitat types: farmland **(a)**, forest **(b)**, grassland **(c)**, Gobi desert **(d)** and mine wasteland **(e)**.

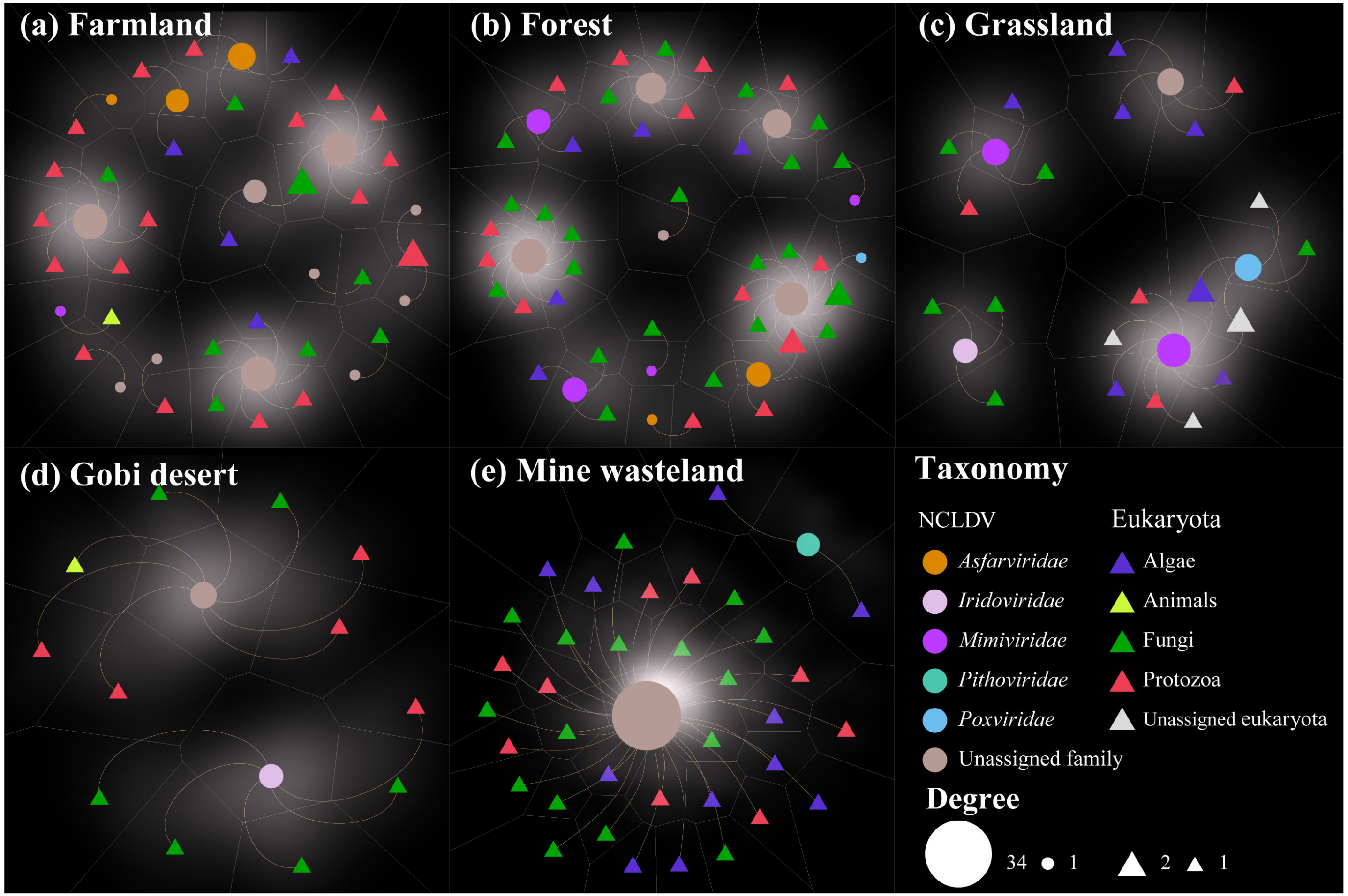

Supplementary Figure 9. The co-occurrence networks of NCLDVs–eukaryotes in different habitat types. Those NCLDV phylotypes and eukaryotic amplicon sequence variants (ASVs) that were present in ≥ 10% of all soil samples for each habitat type were included in our co-

occurrence network analysis. Triangles represent eukaryotic ASVs and circles represent NCLDV phylotypes. The sizes of triangles and circles are proportional to the number of connections. Significant Spearman correlation coefficients ($\rho \geq 0.60$, $P < 0.05$) for NCLDVs-eukaryotes pairs are drawn as edges.

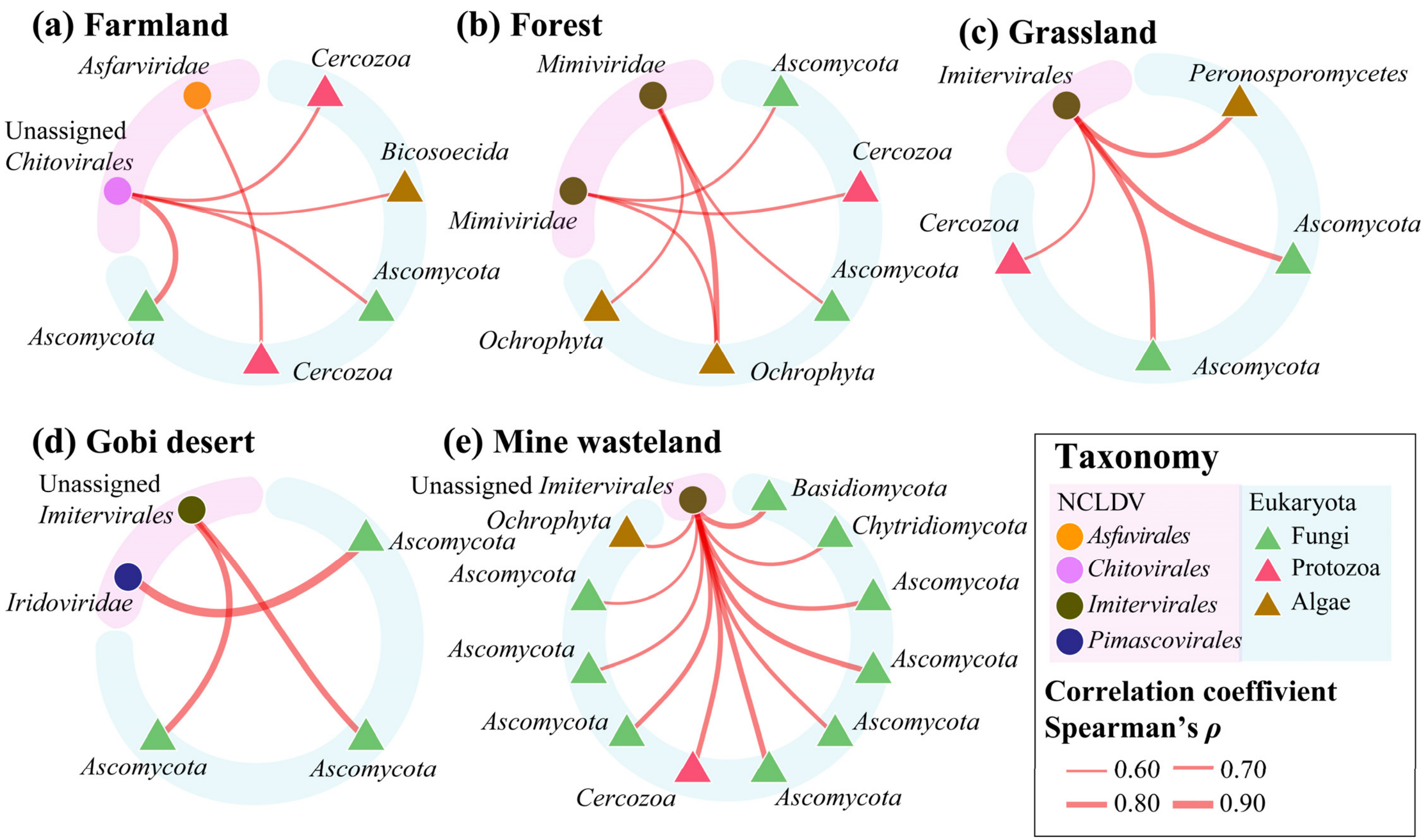

Supplementary Figure 10. The co-occurrence networks of the 14 ubiquitous NCLDVs across four or five habitat types and eukaryotic ASVs. Triangles represent eukaryotic ASVs and circles represent NCLDV phylotypes. Significant Spearman correlation coefficients ($\rho \geq 0.60$, $P < 0.05$) for NCLDV-eukaryote pairs are drawn as edges.

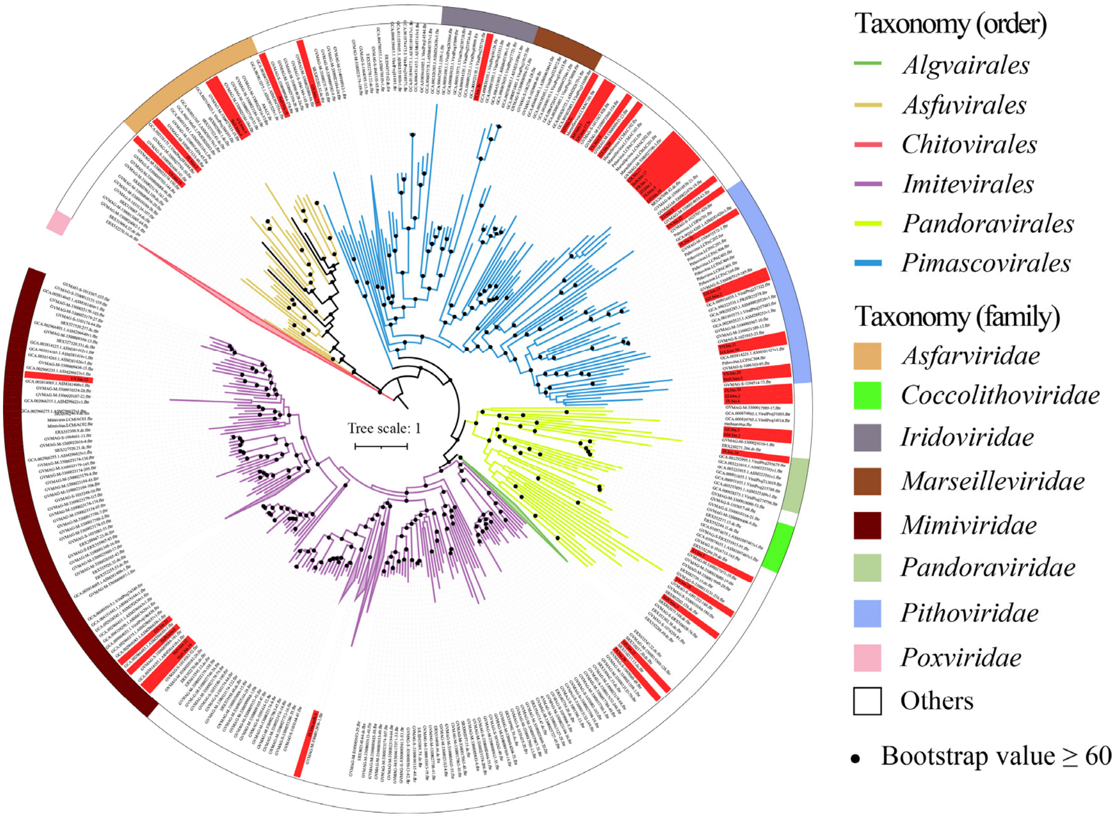

Supplementary Figure 11. The maximum-likelihood phylogenetic tree of giant virus metagenome-assembled genomes (GVMAGs) reconstructed in this study and available in public databases [2]. The tree was built from a concatenated protein alignment of seven marker genes (SFII, RNAPL, PolB, TFIIB, TopoII, A32 and VLTF3) using the model of LG+I+F+G4 and rooted at *Poxvirida*e [2]. Tree branches are colored according to the order-level taxonomic assignment. The GVMAGs recovered from this study are labeled in red background. The outer strip is colored according to the family-level taxonomic assignment. SFII, DEAD/SNF2-like helicase; RNAPL, DNA-directed RNA polymerase alpha subunit; PolB, DNA polymerase family B; TFIIB, transcription initiation factor IIB; TopoII, DNA topoisomerase II; A32, Packaging ATPase; VLTF3, Poxvirus late transcription factor VLTF3.

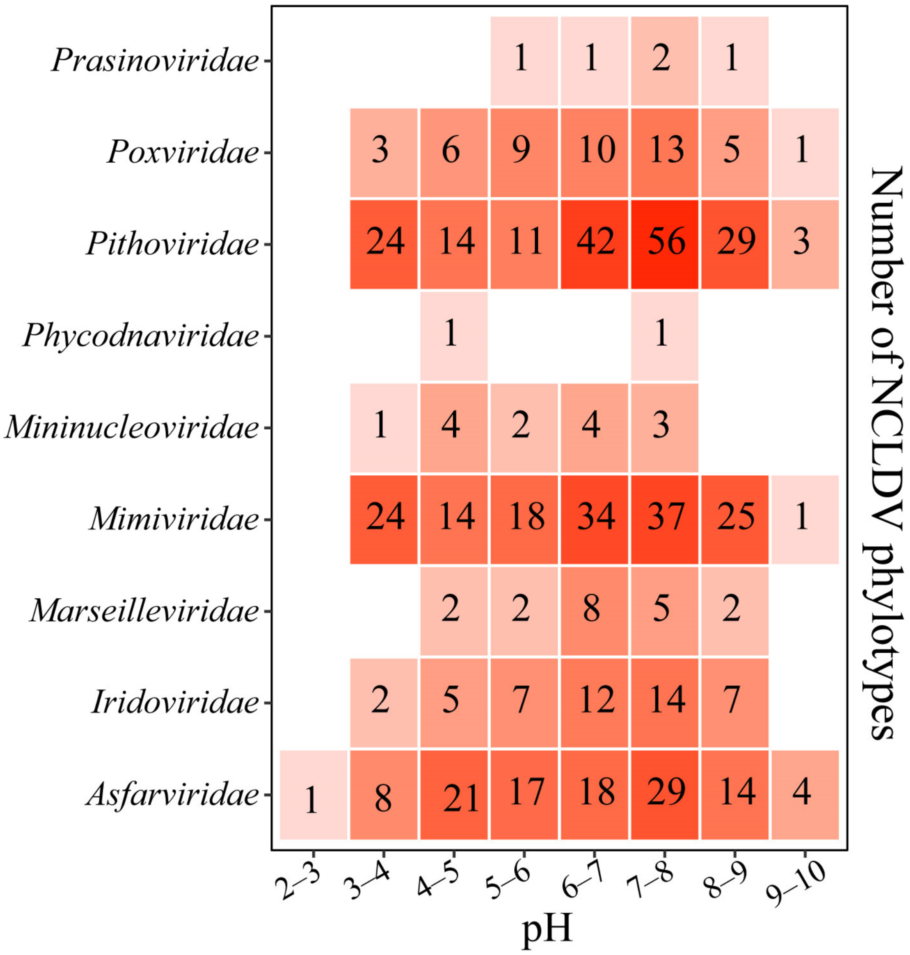

Supplementary Figure 12. The pH-relevant distribution profiles of the numbers of phylotypes belonging to individual NCLDV families. The color intensity of a given grid is proportionate to the number of NCLDV phylotypes belonging to a specific family that can be observed in a given pH range. Given that some phylotypes can occur in a wide range of soil pH, the sum of the numbers shown in the figure is greater than the total number of the NCLDV phylotypes identified in this study.

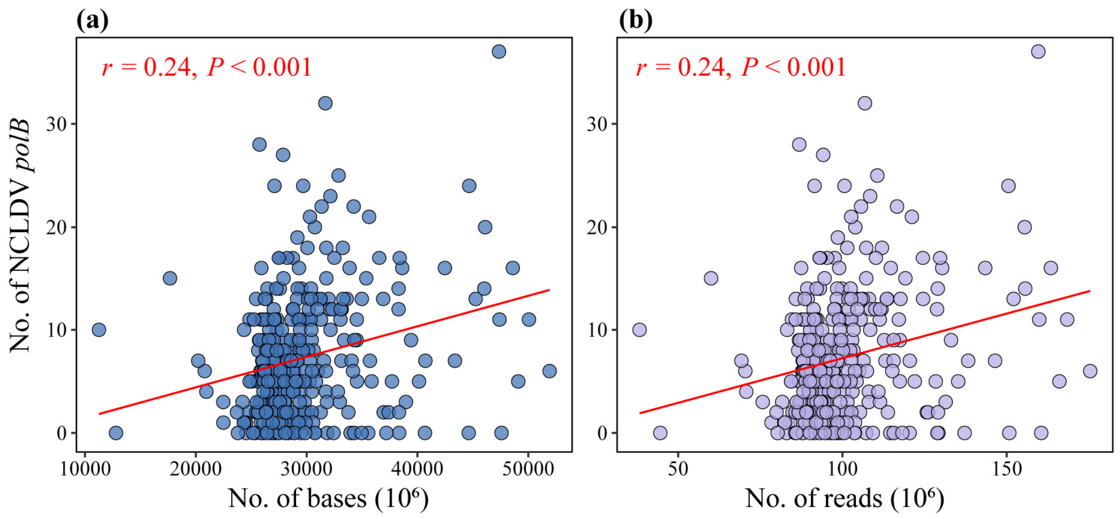

Supplementary Figure 13. Relationships between and the number of NCLDV *polB* genes detected in individual samples and sequencing depth. Each dot in each panel represents one soil sample. The solid red lines represent the linear regression with statistically significant Pearson coefficients.

Supplementary References

1. Endo H, Blanc-Mathieu R, Li Y, Salazar G, Henry N, Labadie K, et al. Biogeography of marine giant viruses reveals their interplay with eukaryotes and ecological functions. Nat Ecol Evol. 2020;4(12):1639–1649.
2. Aylward FO, Moniruzzaman M, Ha AD, Koonin EV. A phylogenomic framework for charting the diversity and evolution of giant viruses. PLoS Biol. 2021;19(10):e3001430.
